# Tripodal tris(hydroxypyridinone) ligands for immunoconjugate PET imaging with ^89^Zr^4+^: comparison with desferrioxamine-B†
†Electronic supplementary information (ESI) available: ^1^H and ^13^C NMR data, reverse phase HPLC and size exclusion HPLC chromatograms, phosphoimages of ITLC plates, *ex vivo* biodistribution data. See DOI: 10.1039/c4dt02978j



**DOI:** 10.1039/c4dt02978j

**Published:** 2014-10-29

**Authors:** Michelle T. Ma, Levente K. Meszaros, Brett M. Paterson, David J. Berry, Maggie S. Cooper, Yongmin Ma, Robert C. Hider, Philip J. Blower

**Affiliations:** a King's College London , Division of Imaging Sciences and Biomedical Engineering , 4th Floor Lambeth Wing , St Thomas’ Hospital , London SE1 7EH , UK . Email: michelle.ma@kcl.ac.uk; b School of Chemistry and Bio21 Molecular Science and Biotechnology Institute , The University of Melbourne , Parkville , Victoria 3052 , Australia; c College of Pharmaceutical Science , Zhejiang Chinese Medical University , Hangzhou , 310053 , People's Republic of China; d King's College London , Institute of Pharmaceutical Science , Franklin Wilkins Building , Stamford St , London SE1 9NH , UK; e King's College London , Division of Chemistry , Britannia House , 7 Trinity St , London SE1 1DB , UK

## Abstract

Due to its long half-life (78 h) and decay properties (77% electron capture, 23% β^+^, *E*_max_ = 897 keV, *E*_av_ = 397 keV, *E*γ = 909 keV, *I*γ = 100%) ^89^Zr is an appealing radionuclide for immunoPET imaging with whole IgG antibodies. Derivatives of the siderophore desferrioxamine-B (H_3_DFO) are the most widely used bifunctional chelators for coordination of ^89^Zr^4+^ because the radiolabeling of the resulting immunoconjugates is rapid under mild conditions. ^89^Zr-DFO complexes are reportedly stable *in vitro* but there is evidence that ^89^Zr^4+^ is released *in vivo*, and subsequently taken up by the skeleton. We have evaluated a novel tripodal tris(hydroxypyridinone) chelator, H_3_CP256 and its bifunctional maleimide derivative, H_3_YM103, for coordination of Zr^4+^ and compared the NMR spectra, and the ^89^Zr^4+^ radiolabeling, antibody conjugation, serum stability and *in vivo* distribution of radiolabelled immunoconjugates with those of H_3_DFO and its analogues. H_3_CP256 coordinates ^89^Zr^4+^ at carrier-free concentrations forming [^89^Zr(CP256)]^+^. Both H_3_DFO and H_3_CP256 were efficiently radiolabelled using [^89^Zr(C_2_O_4_)_4_]^4–^ at ambient temperature in quantitative yield at pH 6–7 at millimolar concentrations of chelator. Competition experiments demonstrate that ^89^Zr^4+^ dissociates from [^89^Zr(DFO)]^+^ in the presence of one equivalent of H_3_CP256 (relative to H_3_DFO) at pH 6–7, resulting largely in [^89^Zr(CP256)]^+^. To assess the stability of H_3_DFO and H_3_YM103 immunoconjugates radiolabelled with ^89^Zr, maleimide derivatives of the chelators were conjugated to the monoclonal antibody trastuzumab *via* reduced cysteine side chains. Both immunoconjugates were labelled with ^89^Zr^4+^ in >98% yield at high specific activities and the labeled immunoconjugates were stable in serum with respect to dissociation of the radiometal. *In vivo* studies in mice indicate that ^89^Zr^4+^ dissociates from YM103-trastuzumab with significant amounts of activity becoming associated with bones and joints (25.88 ± 0.58% ID g^–1^ 7 days post-injection). In contrast, <8% ID g^–1^ of ^89^Zr activity becomes associated with bone in animals administered ^89^Zr-DFO-trastuzumab over the course of 7 days. The tris(hydroxypyridinone) chelator, H_3_CP256, coordinates ^89^Zr^4+^ rapidly under mild conditions, but the ^89^Zr-labelled immunoconjugate, ^89^Zr-YM103-trastuzumab was observed to release appreciable amounts of ^89^Zr^4+^ *in vivo*, demonstrating inferior stability when compared with ^89^Zr-DFO-trastuzumab. The significantly lower *in vivo* stability is likely to be a result of lower kinetic stability of the Zr^4+^ tris(hydroxypyridinone complex) relative to that of DFO and its derivatives.

## Abstract 2

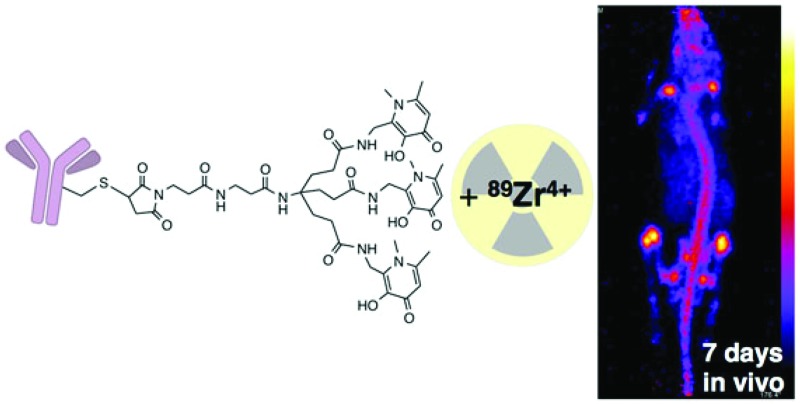
A tris(hydroxypyridinone) chelator coordinates the PET imaging isotope, ^89^Zr^4+^, rapidly and quantitatively under ambient conditions, but a ^89^Zr-labelled tris(hydroxypyridinone)-immunoconjugate is not stable to *in vivo* demetallation.

## Introduction

Antibodies have great utility in the clinic, and can be used without modification as therapeutics or as conjugates in radiotherapy or drug delivery. Currently, the FDA has approved 30 monoclonal antibodies for clinical use, with 12 of these approved for oncological treatments, and hundreds more are in clinical trials.^1^ The ability to image antibody biodistribution and tissue localisation *in vivo* is useful in patient prognosis and dosimetry and in guiding selection of therapeutic regimes and monitoring disease response to antibody-based therapies, and in stratifying patients for clinical trials. Imaging antibody distribution *in vivo* has been mainly achieved with the use of γ-emitting radionuclides, especially ^111^In^2,3^ and ^99m^Tc.^4,5^ In recent years there has been increased interest in using positron emission tomography (PET) to study antibody biodistribution.^6^ The large molecular weight (∼150 kDa) of whole antibodies results in slow accumulation in target tissue, while the lack of domains that mediate clearance and excretion leads to slow blood clearance. Consequently, extended time periods (0.5–7 days) are required for the antibody to clear from non-target tissue and localise at cell receptors in target tissue. The β^+^-emitting isotope ^89^Zr allows these requirements to be met, possessing suitable decay properties (77% electron capture, 23% β^+^, *E*_max_ = 897 keV, *E*_av_ = 397 keV, *E*γ = 909 keV, *I*γ = 100%) and a half-life of 78.5 h.^6–9^

**Chart 1:**
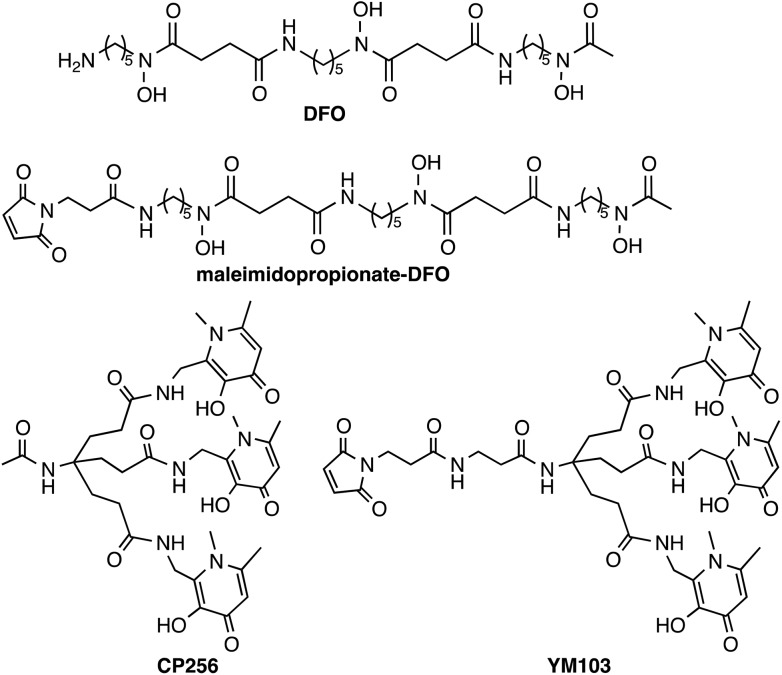
Chelators and bifunctional chelators for ^89^Zr^4+^ coordination.

Zr^4+^ can accommodate up to eight donors in its coordination sphere and the high charge of Zr^4+^ induces a preference for “hard” Lewis acid donor atoms.^8^ The acyclic siderophore ligand desferrioxamine (H_3_DFO)‡
‡To avoid confusion over the oxidation state of the metal and the pH being monitored, we adopt the convention of representing desferrioxamine as “H_3_DFO” – neutral and uncharged – even though the primary amine in water is protonated at pH < 9. (Chart 1) contains three hydroxamate groups. Despite possessing only six donor ligands, H_3_DFO efficiently complexes radiopharmaceutical concentrations of ^89^Zr^4+^ and has been widely used as a bifunctional chelator for ^89^Zr. Linking carboxylate, isothiocyanate or maleimide groups to the terminal amine of H_3_DFO (*e.g.* Chart 1) provides a convenient, stable attachment point for conjugation to amino acids of antibody side chains.^9–14^ Density functional theory calculations indicate that the most energetically favourable coordination geometry of [Zr(DFO)]^+^ consists of an eight-coordinate complex in which two *cis* water molecules and six O atoms of DFO (deprotonated at hydroxamate groups) complex Zr^4+^.^15^ A recent report of an octadentate Zr^4+^ complex that consists of four bidentate *N*-methyl acetohydroxamate ligands coordinated to Zr^4+^ has also provided insight into Zr^4+^ hydroxamate structural chemistry and the stability of such complexes.^16^

H_3_DFO-antibody and other H_3_DFO-protein conjugates labelled with ^89^Zr have been successfully employed in imaging tumours or tumour markers in murine models^15,17–23^ and have demonstrated the ability to image known and unknown tumour lesions in patients in clinical trials.^24–26^ A very recent study has described an octadentate chelator containing four linear hydroxypyridinone groups (each a 1-hydroxy-pyridin-2-one) that is able to coordinate ^89^Zr^4+^ quantitatively.^27^ The resulting complex demonstrates comparable stability to that of [^89^Zr(DFO)]^+^, however to the best of our knowledge, a bifunctional derivative of this complex has not been reported, and the stability of the complex over extended periods of time *in vivo* (>24 h) has not been assessed. Another recent report details the synthesis and ^89^Zr^4+^ labelling of a series of octadentate ligands each containing four hydroxamate ligands.^28^ Linear and macrocyclic derivatives differing in distance between adjacent hydroxamate groups were prepared, and the ability of the new chelators to coordinate radiopharmaceutical concentrations of ^89^Zr^4+^ was demonstrably dependent on the geometry/topology of the ligands. A 36-membered macrocyclic tetra(hydroxamate) species was able to coordinate radiopharmaceutical concentrations of ^89^Zr in >90% radiochemical yield in 30 min, and the resulting complex was more stable than other homologues when subjected to stability studies. The only published reports of novel alternative bifunctional chelators for ^89^Zr describe (i) a linear picolinic acid/methylenephosphonate “mixed” ligand that has been conjugated to trastuzumab,^29^ and very recently (ii) a linear octadentate tetra(hydroxamate) compound, derived from H_3_DFO, that has been attached to a bombesin peptide that targets the gastrin releasing peptide receptor.^30^ The former performed very poorly as a chelator for ^89^Zr (with low radiochemical yields of 8–12%). The latter is able to retain ^89^Zr^4+^ when challenged with excess H_3_DFO over the course of 1 day and appears very promising, although the stability of the complex to demetallation has not been assessed beyond the 24 h time point, or *in vivo*.

Despite the prevalent use of H_3_DFO as a ligand to radiolabel antibodies for clinical and preclinical evaluation, some studies have reported bone uptake suggesting that after prolonged exposure to the *in vivo* milieu, ^89^Zr dissociates from DFO and subsequently accumulates in bone,^12,15,17^ although this is not consistently reported to be a problematic feature of H_3_DFO conjugates.

Hydroxypyridinone ligands and their hexadentate derivatives are extremely effective at sequestering Fe^3+^, Al^3+^ and Ga^3+^,^31–36^ and have been studied for their utility for ^67^Ga^3+^/^68^Ga^3+^ coordination for nuclear medicine applications.^37,38^ We previously reported that a tris(hydroxypyridinone) ligand, H_3_CP256^33^ and its bifunctional derivative, H_3_YM103 (Chart 1), each incorporating three 1,6-dimethyl-3-hydroxypyridin-4-one groups, have outstanding properties as chelators of the radioisotopes ^67^Ga^3+^ and ^68^Ga^3+^ at radiopharmaceutical concentrations.^38^ The bifunctional chelator H_3_YM103, which contains a maleimide group, was originally developed to allow facile site-specific modification of proteins through engineered cysteine residues. The tris(hydroxypyridinone) ligands are efficient at extremely low concentrations of chelator, and the resulting complexes and bioconjugates are stable under *in vivo* biological conditions. With its six oxygen donors, we speculated that by analogy to H_3_DFO, which coordinates to Fe^3+^, Ga^3+^ and Zr^4+^, H_3_CP256 and H_3_YM103 might coordinate to ^89^Zr^4+^ under conditions appropriate for convenient labelling of proteins, and that the resulting complexes might be sufficiently stable for PET imaging with antibodies, offering an alternative to H_3_DFO. We note that similar to DFO, H_3_CP256 is only capable of providing six oxygen donors, and cannot coordinatively saturate Zr^4+^ that can bind up to eight ligand atoms, however prior to synthesising tetra(hydroxypyridinone) ligands, it was instructive to characterise and define the behaviour of [^89^Zr(CP256)]^+^ and investigate whether a multidentate ligand based on three 1,6-dimethyl-3-hydroxypyridin-4-one groups can coordinate ^89^Zr^4+^.

Herein we describe the ^89^Zr^4+^ radiolabelling of H_3_CP256 and its bifunctional analogue H_3_YM103 conjugated to the monoclonal antibody trastuzumab. Trastuzumab is currently an approved clinical metastatic breast cancer therapeutic. It binds to the human epidermal growth factor receptor 2 (HER2) and inhibits the over-proliferative effects of HER2 overexpression. Trastuzumab conjugates of H_3_DFO labelled with ^89^Zr have been successfully employed to image HER2 positive tumours and assess HER2 expression levels.^24,39,40^ Therefore native trastuzumab was utilised here as a model antibody to assess the properties of a ^89^Zr-labelled tris(hydroxypyridinone) conjugate. The stability and biodistribution of the immunoconjugate labelled with ^89^Zr *via* the novel tris(hydroxypyridinone) ligand were compared with those of a trastuzumab immunoconjugate labelled with ^89^Zr *via* a H_3_DFO ligand. Additionally, we report NMR spectroscopic data for [Zr(DFO)]^+^, formed *in situ* from [Zr(acac)_4_] and H_3_DFO. Despite the widespread use of bifunctional H_3_DFO ligands for ^89^Zr labelling, there are no reported spectroscopic data available.

## Results

### H_3_CP256 complexation with Zr^4+^

**Fig. 1:**
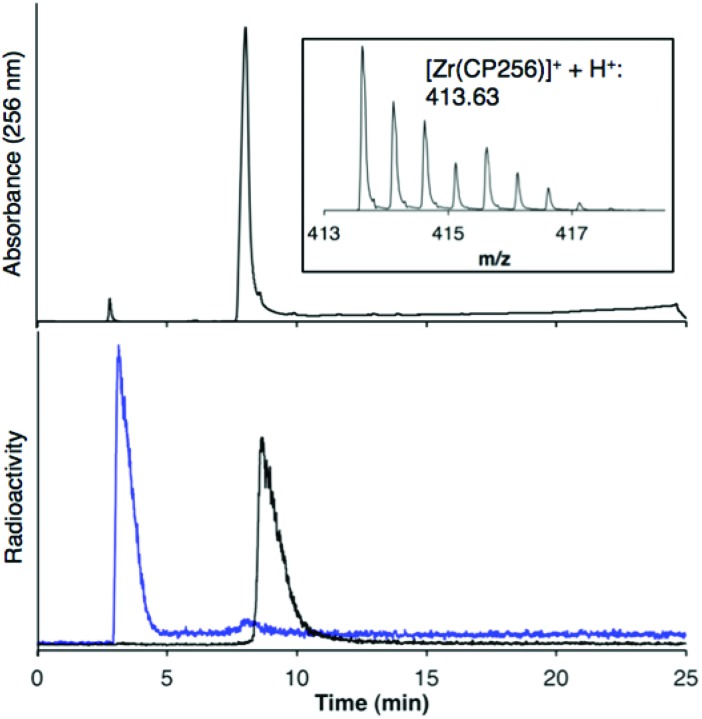
LCMS of [Zr(CP256)]^+^. Top: HPLC chromatogram with UV detection at *λ*_256_ of [Zr(CP256)]^+^ and corresponding MS signal (inset) of the fraction eluting at 8.0 min; Bottom: HPLC chromatogram of [^89^Zr(CP256)]^+^ (8.61 min) (black trace) and [^89^Zr(ox)_4_]^4–^ (blue trace) with radio-scintillation detection. Difference in retention times for the same species between UV and radio-scintillation HPLC chromatograms is a result of the configuration of the detectors in series.

When one equivalent of [Zr(acac)_4_] was added to a solution of H_3_CP256 in methanol, a Zr^4+^ coordinated complex of CP256 was formed, and the mass spectrum indicated that a mononuclear Zr^4+^ complex with an *m*/*z* value (413.61, *z* = 2+, 100% of the normalised spectrum and 826.22, *z* = 1+, 22.5%) corresponding to [Zr(CP256)]^+^ ({[Zr(C_36_H_46_N_7_O_10_)] + H}^2+^ calculated = 413.62 *m*/*z*, [Zr(C_36_H_46_N_7_O_10_)]^+^ calculated = 826.24) (Fig. 1, top inset) was present. The mass spectrum did not reveal significant populations of any other species (*e.g.* oligomers or complexes with ligand-to-metal stoichiometric ratios other than 1 : 1) with relative abundance ≥5% (of the normalised spectrum). In the monocationic complex [Zr(CP256)]^+^, the three hydroxyl substituents of the hydroxypyridinone rings are deprotonated. Under the LCMS conditions employed here, the complex possessed a retention time of 8.01 min (Fig. 1, top chromatogram), which is longer than that of H_3_CP256 (7.47 min), and no other signals in the chromatogram with a corresponding characteristic ^nat^Zr mass spectral isotope signature were detected (with the exception of the signal at 2.78 min that corresponds to elution of excess ^nat^Zr^4+^).

**Fig. 2:**
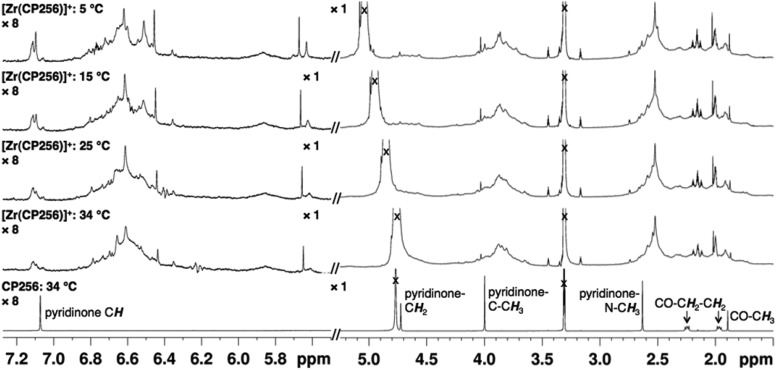
^1^H VT NMR spectra in methanol-*d*_4_ of H_3_CP256 (bottom) and [Zr(CP256)]^+^ demonstrating high fluxionality of the complex. Intensity of signal >5.5 ppm is magnified (×8) relative to region <5.5 ppm. ^1^H protons of acetylacetonate ligand have exchanged with deuterons.

The ^1^H NMR spectral features of a sample of [Zr(CP256)]^+^ in methanol-*d*_4_ are extremely broad (Fig. 2), presumably a consequence of fluxionality within the complex. Acquiring spectra at 5 °C resulted in sharpening of some spectral features, but overall, resonances could not be definitively assigned. Coordination of Zr^4+^ resulted in a significant local effect on shifts and broadening of hydroxypyridinone ring and ring substituent resonances, but had a less marked effect on resonances of the tripodal framework –C*H*_2_–C*H*_2_– protons. Zr^4+^ binding to the aromatic ring resulted in significantly increased shielding of hydroxypyridinone C*H*, N–C*H*_3_ and C*H*_3_ protons, but substantial deshielding of the hydroxypyridinone C*H*_2_ protons. Aqueous samples acquired in deuterium oxide resulted in ^1^H NMR spectra with similar line shapes and chemical shifts (Fig. S1, ESI†).

**Fig. 3:**
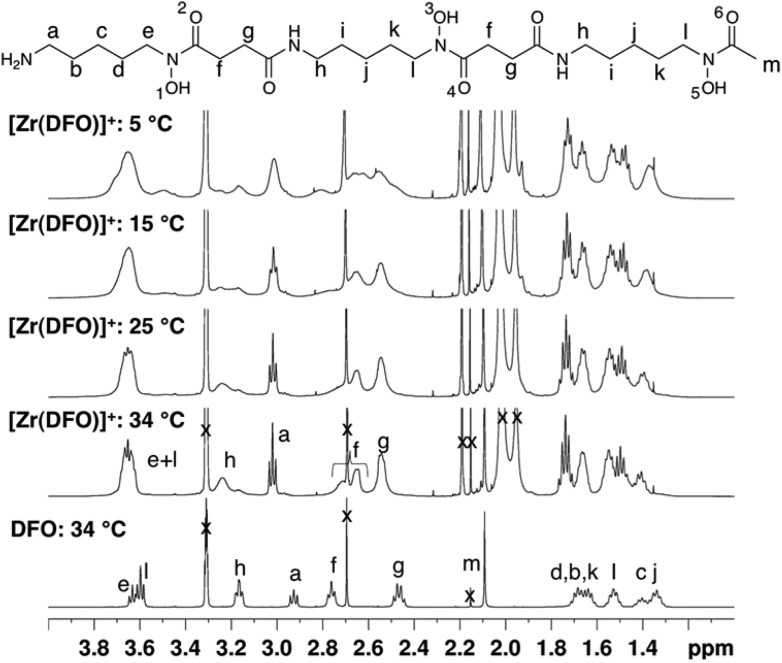
Variable temperature ^1^H NMR spectra in of DFO and [Zr(DFO)]^+^ in methanol-*d*_4_. Additional resonances in [Zr(DFO)]^+^ spectra result from excess [Zr(acac)_4_], acac, mesylate ion, acetone and solvent. In the case where chemically inequivalent atoms have the same label, the similar chemical environment of the atoms resulted in coincident resonances at 500 MHz for ^1^H NMR, and coincident, or almost coincident resonances at 100 MHz (on a Bruker Avance 400 spectrometer) for ^13^C NMR.

**Fig. 4:**
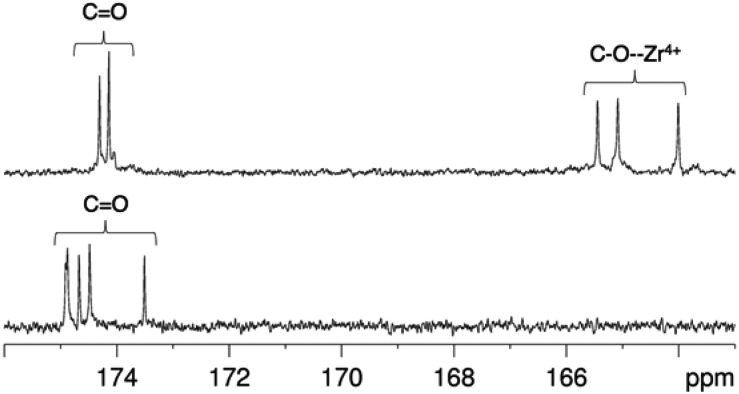
^13^C NMR spectra in of [Zr(DFO)]^+^ (top) and DFO (bottom) in methanol-*d*_4_ at 25 °C, demonstrating the shift in ^13^C=O resonances upon Zr^4+^ coordination.

In contrast, the ^1^H NMR spectra of H_3_DFO and [Zr(DFO)]^+^ acquired in methanol-*d*_4_ exhibited distinct signals (Fig. 3), and assignments were made from COSY and HSQC spectra. The ^1^H NMR spectrum of H_3_DFO was consistent with previously reported data.^39^ For [Zr(DFO)]^+^ at low temperatures, resonances were broadened compared to those at higher temperatures, and at all temperatures measured, the broadness of some methylene resonances prohibited observation of coupling constants and splitting patterns. At both 400 and 500 MHz, ^1^H signals from chemically inequivalent methylene groups in similar environments are coincident. For both the free ligand and the Zr^4+^ coordinated ligand, even though the two methylene groups both labeled h in Fig. 3 are chemically distinct, the protons of each group resonate at the same chemical shift. It is expected that coordination of Zr^4+^ would result in formation of enantiomers and other isomers in the case of either a six, seven or eight coordinate environment. DFT structures for these alternative environments have previously been calculated and described.^15^ Broad line shapes for [Zr(DFO)]^+^ obscure separation of resonances of geminal protons that are necessarily diastereotopic and chemically inequivalent as a result of the formation of a large chelate ring upon Zr^4+^ coordination (with the exception of protons a–e). Resonances become increasingly broad at lower temperatures. ^13^C NMR spectra were acquired for both H_3_DFO and [Zr(DFO)]^+^ (Table 1). As was the case with the ^1^H NMR spectra, ^13^C resonances are coincident in some instances where two chemically inequivalent methylene groups are in a similar chemical environment. Upon Zr^4+^ coordination, resonances for hydroxamate ^13^C=O groups underwent marked shifts to lower frequencies (Fig. 4). The ^1^H resonance for methylene group a moved to higher chemical shift, whilst the corresponding ^13^C methylene signal did not shift significantly (40.7 ppm in H_3_DFO to 40.6 ppm in [Zr(DFO)]^+^). ^1^H and ^13^C NMR spectra of [Zr(DFO)]^+^ were also recorded in deuterium oxide and similar spectra were obtained (Fig. S2 and S3, ESI†), thus demonstrating that the same complex exists in methanol and water. Under the deuterated solvent conditions employed, any coordinated solvent or water molecules could not be observed, and there was no evidence to suggest that acetylacetonate present in solution coordinates to [Zr(DFO)]^+^.

**Table 1:** ^13^C NMR assignments of H_3_DFO and [Zr(DFO)]^+^ in methanol-d_4_. For labeling scheme, refer to Fig. 3

Assignment	DFO	[Zr(DFO)]^+^
a	40.7	40.6
b, c, d, i, j, k	24.3, 24.87, 24.90, 27.0, 27.3, 28.0 30.0	23.4, 23.77, 23.85, 27.5, 27.9, 28.31, 28.35, 29.00, 29.09.
e	48.4	51.2
f	28.7, 28.9	26.4
g	31.2, 31.4	32.1, 32.5
h	40.3	39.5, 40.4
l	48.6	51.5
m	20.2	17.0
C=O	173.5, 174.5, 174.7, 174.88, 174.91	164.0, 165.1, 165.5, 174.1, 174.3

### Radiolabelling H_3_CP256 with ^89^Zr^4+^

Addition of [^89^Zr(ox)_4_]^4–^ (0.5 MBq) to a solution of H_3_CP256 (1 mM, 20 μL) at pH 6.5 resulted in formation of a single radiolabelled species, [^89^Zr(CP256)]^+^, which possessed the same LCMS retention time as the nonradioactive complex when corrected for delay between detectors (Fig. 1, bottom chromatogram – black trace) and demonstrated significantly greater retention on the C18 reverse-phase column than [^89^Zr(ox)_4_]^4–^ (Fig. 1, bottom chromatogram – blue trace).

**Fig. 5:**
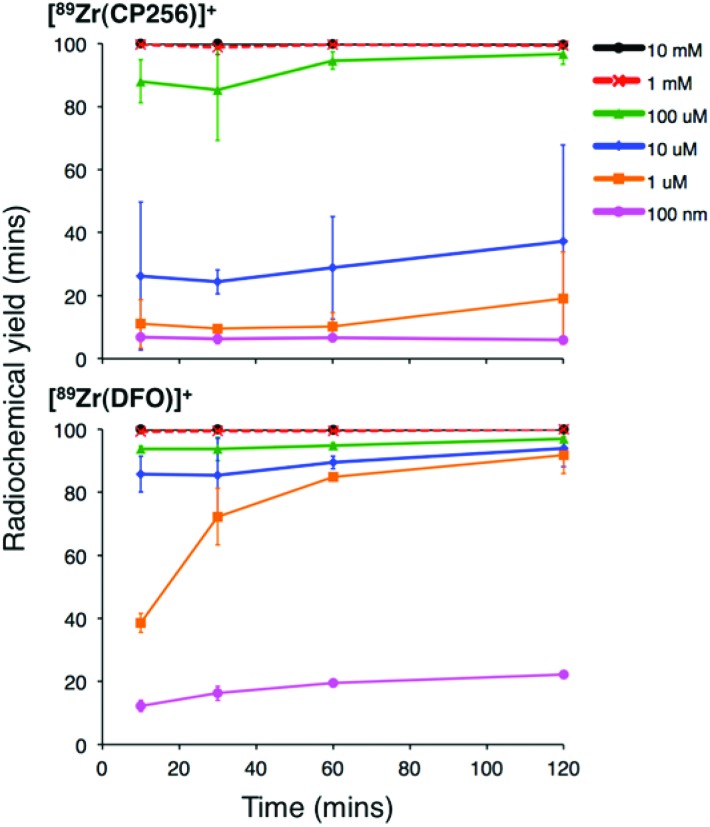
Radiochemical yield of [^89^Zr(CP256)]^+^ (top) and [^89^Zr(DFO)]^+^ (bottom) at different concentrations of chelator over the course of two hours. Error bars correspond to one standard deviation.

High specific activity is often crucial when synthesising radiotracers for targeted molecular imaging. High concentrations of unlabelled targeting agent lead to receptor “blocking” and therefore compromised image interpretation. To assess the ability of H_3_CP256 to complex ^89^Zr^4+^ at dilute concentrations of chelator, ^89^Zr^4+^ (∼0.2 MBq, 1 μL) was added to increasingly dilute solutions of H_3_CP256 (10 μL, pH 6.5, 100 μM ammonium acetate). Similar experiments were also undertaken using H_3_DFO, to compare the relative abilities of H_3_DFO and H_3_CP256 to complex radiochemical concentrations of ^89^Zr^4+^. Aliquots of these reaction solutions were analysed by ITLC at 10 min, 30 min, 60 min and 120 min reaction time, to determine the radiochemical yield (Fig. 5). For both chelators, quantitative coordination of ^89^Zr^4+^ was only observed at the highest and second highest concentrations measured, 10 mM and 1 mM (>99% radiochemical yield at all time points). A 10-fold lower concentration of both chelators (100 μM) resulted in reasonably high radiochemical yields – between 94 and 96% (which would typically be regarded as adequate for use in imaging without further purification) after 60 min at ambient temperature. Lower concentrations of H_3_CP256 (10 μM–100 nM) yielded less than 20% of [^89^Zr(CP256)]^+^ at all time points. Lower concentrations of H_3_DFO resulted in consistently higher yields compared to H_3_CP256. For example, for [H_3_DFO] = 10 μM at *t* = 60 min, the radiochemical yield was 90%, and for [H_3_DFO] = 1 μM, *t* = 60 min, the radiochemical yield was 75%.

### Competition studies

**Fig. 6:**
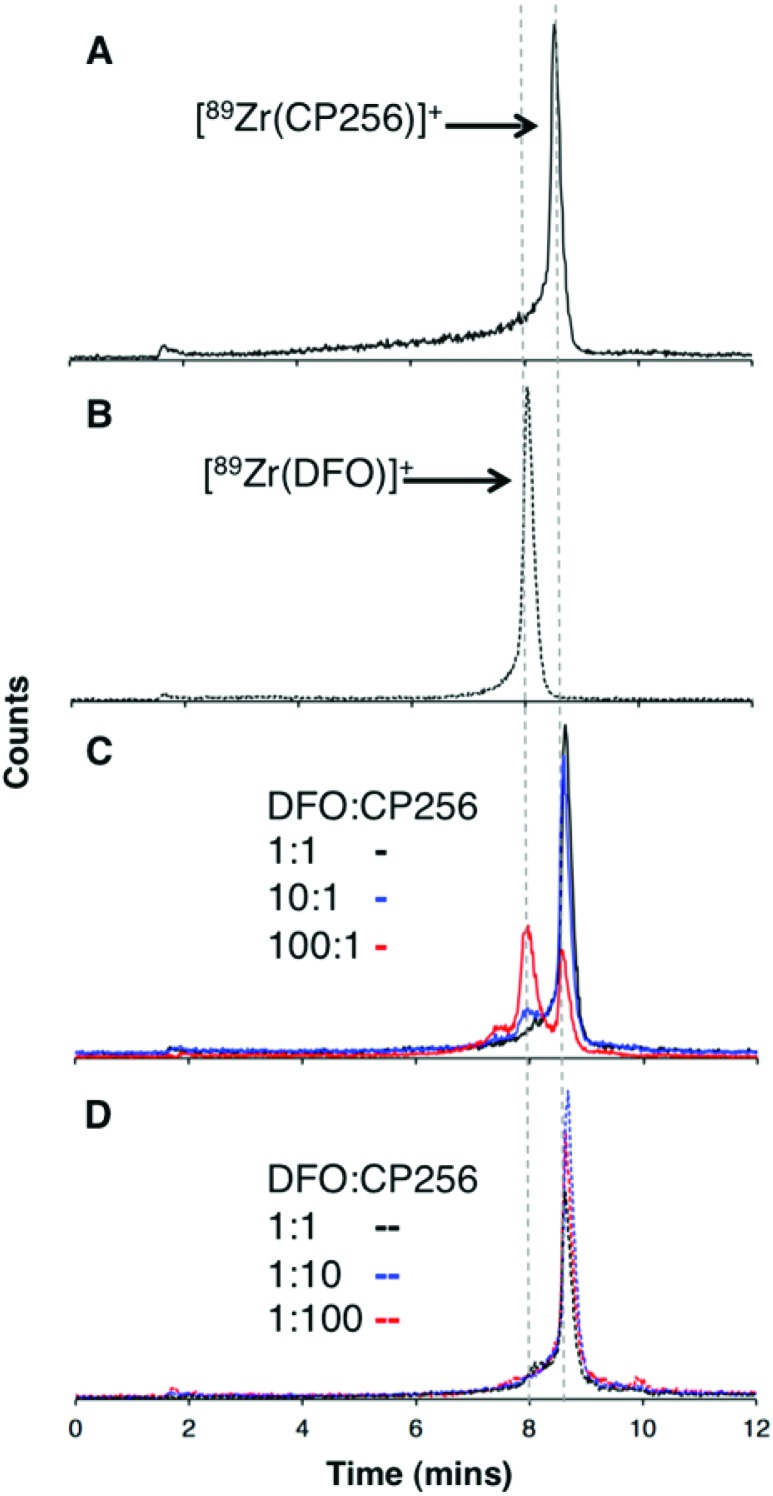
Reverse phase radiochromatogram of (A) [^89^Zr(CP256)]^+^ (black) and (B) [^89^Zr(DFO)]^+^ (dotted line); (C) A solution of [^89^Zr(CP256)]^+^ incubated with H_3_DFO ligand (H_3_CP256–H_3_DFO = 1 : 1, black line); (H_3_CP256–H_3_DFO = 1 : 10, blue line); (H_3_CP256–H_3_DFO = 1 : 100, red line); in all experiments, final [H_3_CP256] = 500 μM. (D) A solution of [^89^Zr(DFO)]^+^ incubated with H_3_CP256 ligand (H_3_DFO–H_3_CP256 = 1 : 1, black line); (H_3_DFO–H_3_CP256 = 1 : 10, blue line); (H_3_DFO–H_3_CP256 = 1 : 100, red line); in all experiments, final [H_3_DFO] = 500 μM.

Competition studies were undertaken, in which increasingly concentrated solutions of H_3_DFO were added to H_3_CP256 solutions containing [^89^Zr(CP256)]^+^. Solutions of H_3_CP256 were also added to H_3_DFO solutions containing [^89^Zr(DFO)]^+^. The RP-HPLC retention times of [^89^Zr(CP256)]^+^ and [^89^Zr(DFO)]^+^ differ under the conditions employed here, so RP-HPLC was used to distinguish the products of these reactions (Fig. 6A and B).

Addition of 1 equivalent of H_3_DFO (10 μL, 1 mM H_3_DFO, 100 μM ammonium acetate) to a solution containing 1 equivalent of H_3_CP256 (10 μL, 1 mM H_3_CP256) and [^89^Zr(CP256)]^+^ (∼0.4 MBq) did not result in appreciable transmetallation of ^89^Zr^4+^ from CP256 to DFO (Fig. 6C). In contrast, addition of 1 equivalent of H_3_CP256 to 1 equivalent of H_3_DFO containing [^89^Zr(DFO)]^+^ (∼0.4 MBq) resulted in dissociation of ^89^Zr^4+^ from DFO, and coordination to CP256. There was no discernable difference in the ratio of products at 1 h and 12 h, indicating that equilibrium was reached by 1 h at ambient temperature. Addition of 10 equivalents of H_3_DFO (10 μL, 10 mM H_3_DFO) to 1 equivalent of H_3_CP256 (10 μL, 1 mM H_3_CP256) containing [^89^Zr(CP256)]^+^ (∼0.4 MBq) resulted in <15% dissociation of ^89^Zr^4+^ from CP256 (>85% remains bound) (Fig. 6C). Addition of 100 equivalents of H_3_DFO (10 μL, 100 mM H_3_DFO) to 1 equivalent of H_3_CP256 (10 μL, 1 mM H_3_CP256) containing [^89^Zr(CP256)]^+^ (∼0.4 MBq) resulted in ∼65% formation of [^89^Zr(DFO)]^+^ with ∼20% ^89^Zr^4+^ remaining bound to CP256 (Fig. 6C). The remaining ∼15% of activity was associated with a small “shoulder” peak centered at 7.45 min that could not be resolved from [^89^Zr(DFO)]^+^ (8.03 min) and was not present in either of the chromatograms of [^89^Zr(DFO)]^+^ or [^89^Zr(CP256)]^+^. This is presumably an intermediate in the transchelation of ^89^Zr^4+^ from CP256 to DFO. Addition of either 10 equivalents or 100 equivalents of H_3_CP256 to 1 equivalent of H_3_DFO containing [^89^Zr(DFO)]^2+^ (∼0.4 MBq) resulted in complete transmetallation of ^89^Zr^4+^ from DFO to CP256 (Fig. 6D). Both sets of reactions were also duplicated in phosphate buffer (pH 7.4, 0.1 M) for solutions containing competing chelator at 1 mM and 10 mM concentrations, resulting in identical outcomes.

To verify that these reaction products were in fact equilibrium products (as opposed to kinetic products), 1 equivalent of H_3_CP256 (10 μL, 1 mM H_3_CP256) was added to 10 equivalents of H_3_DFO (10 μL, 10 mM H_3_DFO) containing [^89^Zr(DFO)]^2+^, and in a separate reaction 1 equivalent of H_3_CP256 (10 μL, 1 mM H_3_CP256) was added to 100 equivalents of H_3_DFO (10 μL, 100 mM H_3_DFO) containing [^89^Zr(DFO)]^2+^. The ratios of products were the same as those previously observed for the same ratio of chelators (Fig. S4, ESI†). Thus the order in which the solutions are labelled in these experiments is inconsequential as the reactions are under thermodynamic control.

Lastly, it is conceivable that rapid re-equilibration takes place on the C18 reverse phase HPLC column under the acidic mobile phase conditions (0.1% trifluoroacetic acid, pH 1–2). Attempts to separate reaction products by HPLC at neutral pH were unsuccessful. Whilst the *R*_f_ values of [^89^Zr(CP256)]^+^ (*R*_f_ = 0) and [^89^Zr(DFO)]^+^ (*R*_f_ = 0–0.5) rendered quantitative ITLC analysis using the described system impossible (mobile phase 0.1 M sodium citrate, pH 5.5), the differences in the overall appearance of the ITLC plates following visualisation using a phosphoimager could qualitatively identify which species predominated in solution. [^89^Zr(DFO)]^+^ resulted in “streaking” whereas [^89^Zr(CP256)]^+^ resulted in a single, well-resolved “spot” (Fig. S5a and S5b, ESI†). To this end, a solution of [^89^Zr(DFO)]^+^ ([H_3_DFO] = 1 mM, 5 μL) was added to solutions of H_3_CP256 (([H_3_DFO] = 100 μM or 1 mM, 5 μL) to give two solutions in which the ratio of H_3_CP256–H_3_DFO = 0.1 or 1. In the case of the solution where H_3_CP256–H_3_DFO = 1, visualisation of the ITLC plate revealed that only a single “spot” was present, indicative of the predominance of [^89^Zr(CP256)]^+^ in solution (Fig. S5c†). In the case of the solution where H_3_CP256–H_3_DFO = 0.1, a single spot with very faint streaking was observed, again indicating the predominance of [^89^Zr(CP256)]^+^ in solution (Fig. S5d†). This qualitative ITLC evidence corroborated the observation that H_3_CP256 is able to successfully compete for ^89^Zr^4+^ binding, at concentrations equal to, or 0.1–0.01 fold lower than that of DFO.

### Stability of DFO and CP256 complexes of ^89^Zr^4+^ in the presence of Fe^3+^

**Fig. 7:**
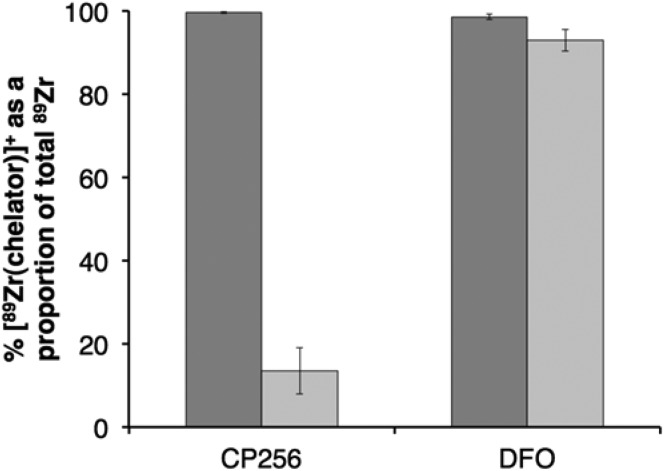
Stability of ^89^Zr^4+^ complexes of H_3_CP256 (left) and H_3_DFO (right): (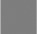) % radiochemical yield of [^89^Zr(chelator)]^+^ at 100 μM concentration of chelator; (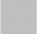) % intact [^89^Zr(chelator)]^+^ as a proportion of total ^89^Zr^4+^ (bound + dissociated) after 20 min incubation with 1 mM Fe^3+^. Error bars correspond to one standard deviation.

The stabilities of [^89^Zr(CP256)]^+^ and [^89^Zr(DFO)]^+^ were assessed in the presence of Fe^3+^ ions. Solutions of [^89^Zr(CP256)]^+^ and [^89^Zr(DFO)]^+^ containing a 10-fold excess of Fe^3+^ over the respective chelator were compared by ITLC, to determine the relative amount of ^89^Zr^4+^ that dissociates from the chelator upon addition of Fe^3+^ followed by incubation at ambient temperature for 20 min (Fig. 7). In the case of H_3_DFO, addition of Fe^3+^ ([Fe^3+^] = 1 mM in final solution, [H_3_DFO] = 100 μM) did not result in more than 7% dissociation of ^89^Zr^4+^ from DFO, however, in the case of H_3_CP256, addition of Fe^3+^ ([Fe^3+^] = 1 mM in final solution, [H_3_CP256] = 100 μM), resulted in dissociation of almost 86% of ^89^Zr^4+^ from the ligand. This was not observed at measured higher concentrations of H_3_CP256 – when [H_3_CP256] = [Fe^3+^] = 1 mM, any measured dissociation of ^89^Zr^4+^ from H_3_CP256 in the timeframe of the experiment (approximately 3%) was not substantial.

### Synthesis and radiolabeling of BFC-trastuzumab immunoconjugates

The monoclonal antibody (Mab), trastuzumab, was chosen as a model antibody for assessing the serum stability and *in vivo* stability of ^89^Zr-coordinated immunoconjugates. Following reduction of Mab disulfide bonds with tris(2-carboxyethyl)phosphine (TCEP) to produce free thiol groups, the bifunctional chelators (BFC), H_3_YM103 and a maleimidopropionate-desferrioxamine derivative were conjugated to trastuzumab *via* the maleimide functional groups. After 30 min reaction time, the immunoconjugates were separated from unreacted BFC using a NAP5 Sephadex size exclusion column and analysed using size exclusion chromatography and ESI-MS. Both the H_3_DFO and H_3_YM103 immunoconjugates possessed the same retention time as unmodified trastuzumab antibody, demonstrating that reduction with TCEP and subsequent conjugation did not result in dissociation or fragmentation of the antibody in solution.

Following TCEP reduction and conjugation and under the conditions utilised for ESI-MS analysis the heavy and light chains of the antibody dissociated in the gas phase, giving rise to separate signals. In contrast, the ESI mass spectrum of non-TCEP-treated unmodified trastuzumab antibody revealed that unmodified/unreduced trastuzumab did not dissociate into heavy and light chain fragments. To facilitate analysis and enable comparison between conjugated and unconjugated trastuzumab antibody fragments, a sample of trastuzumab was treated with TCEP and separated using a NAP5 Sephadex cation exchange column. This sample was immediately subjected to ESI-MS analysis. Subsequent deconvolution of the spectrum revealed the molecular weight of reduced trastuzumab fragments that separated in the gas phase – in this case, the unconjugated light, heavy and heavy-light chains. The molecular weight of each observed fragment of reduced trastuzumab and the H_3_DFO and H_3_YM103 trastuzumab immunoconjugates is listed in Table 2.

**Table 2:** Summary of immunoconjugate peaks observed by ESI-MS

Assignment	*m*/*z* ^*a*^
Trastuzumab	
Light chain	23 439
Heavy chain	50 594 (50 757)
Light-heavy chain	74 031 (74 194)

H_3_YM103-trastzumab	
Light chain	23 439
Light chain + H_3_YM103	24 360
Heavy chain	50 594 (50 755)
Heavy chain + H_3_YM103	51 519 (51 676)
Heavy chain + 2(H_3_YM103)	52 439 (52 596)
Heavy chain + 3(H_3_YM103)	53 356 (53 518)
Light-heavy chain	74 031 (74 194)
Light-heavy chain + H_3_YM103	74 954 (75 118)
Light-heavy chain + 2(H_3_YM103)	75 877 (76 038)

H_3_DFO-trastuzumab	
Light chain + 1(H_3_DFO)	24 151
Heavy chain + 3(H_3_DFO)	52 730 (52 892)
Light-heavy chain + 2(H_3_DFO)	75 466 (75 617)

^*a*^Signal in brackets due to additional hexose unit in variably glycosylated heavy chain.

ESI-MS analysis of trastuzumab immunoconjugates, followed by deconvolution of the subsequent spectrum, revealed that four to eight H_3_DFO groups were attached to each trastuzumab antibody. In the case of a species conjugated to eight H_3_DFO groups, three H_3_DFO chelators were attached to each heavy chain and one attached to each light chain. There were no signals corresponding to unconjugated light chain, or a heavy chain conjugated to less than three H_3_DFO groups. In the case of a species conjugated to four H_3_DFO groups, the corresponding mass spectral signals indicated that two H_3_DFO groups were attached to each associated heavy-light chain fragment. No unconjugated fragments were observed in this spectrum. For H_3_YM103-trastuzumab, the mass spectrum indicated that a mixture of species was present, varying in the number of attached H_3_YM103 groups. From the relative intensities of signals in the ESI mass spectrum, it appeared that only a small fraction of light chain fragments were conjugated to a single H_3_YM103 and the strongest light chain signal corresponded to unconjugated light chain. Signals observed for the heavy chain corresponded to heavy chain conjugated to zero to three H_3_YM103 groups. Signals were also observed for associated heavy-light chain fragments, conjugated to zero, one and two H_3_YM103 groups, all of comparable total ion count intensity.

It is also noteworthy that each heavy chain signal and each heavy-light chain signal in the spectra of both immunoconjugates and reduced trastuzumab was accompanied by an additional signal with a difference of 162–164 Da. This signal corresponds to one additional hexose unit suggesting the presence of differently glycosylated forms of the heavy chain of trastuzumab in the gas phase.

Size exclusion HPLC measurements were used to determine the radiochemical yield of ^89^Zr-labelled immunoconjugates, and as the radiochemical yield and purity were >98% at the specific activities listed below, no further purification was undertaken. Radiolabelled ^89^Zr-YM103-trastuzumab was obtained with a radiochemical yield of 98.7% and a specific activity of 55 MBq mg^–1^ immunoconjugate. Radiolabelled ^89^Zr-DFO-trastuzumab was obtained with a radiochemical yield of 98.3% and a specific activity of 91 MBq mg^–1^ immunoconjugate.

Antibody-antigen dissociation constants (*K*_d_) for ^89^Zr-YM103-trastuzumab and ^89^Zr-DFO-trastuzumab were determined in a competitive binding assay using HCC1954 HER2-positive cells. For ^89^Zr-YM103-trastuzumab, *K*_d_ = 9.4 nM (3.28–15.61 nM, 95% confidence interval) and for ^89^Zr-DFO-trastuzumab, *K*_d_ = 2.6 nM (0.66–4.49 nM, 95% confidence interval), demonstrating that both labelled immunoconjugates retain their affinity for the HER2 receptor (*K*_d_ = 5 nM).^41^

### Serum stability studies

To assess the stability of the ^89^Zr-labelled immunoconjugates in a biological milieu, ^89^Zr-DFO-trastuzumab and ^89^Zr-YM103-trastuzumab were incubated in fresh serum. The samples were incubated at 37 °C in a 5% CO_2_ atmosphere for seven days, and size exclusion chromatography was used to assess the stability of the immunoconjugates over this time course. As the mobile phase (100 mM phosphate buffer, pH 7.4) contained EDTA (2 mM), the majority of dissociated ^89^Zr^4+^ that is coordinated to solvent or electrolyte, or weakly bound to serum proteins, is complexed by EDTA and contributes to a signal with a retention time of 11–12 min. This was confirmed experimentally by incubating a solution of serum with [^89^Zr(ox)_4_]^4–^ for 48 h – see Fig. S6, ESI.† The chromatogram exhibited signals at 11.40 min (57% of total radioactivity) with a shoulder at 11.85 min (30%), and a small signal at 8.80 min (13%). The former signals correspond to [^89^Zr(EDTA)] or small molecular weight ^89^Zr complexes. The latter signal likely corresponds to ^89^Zr bound to a serum protein with molecular weight <150 kDa (possibly transferrin^41^) (Fig. S6†). After incubation in serum for 2 days, both ^89^Zr-DFO-trastuzumab and ^89^Zr-YM103-trastuzumab were intact, and at 7 days, >95% of ^89^Zr remained associated with the mAb fraction (Fig. S6†). Over this time, some aggregation of ^89^Zr-YM103-trastuzumab occurred in the serum solution, as evidenced by the appearance of shoulder peaks at lower retention times. In the course of these studies, it was also observed that neither native (unmodified) trastuzumab nor reduced trastuzumab (unconjugated to chelators) coordinated ^89^Zr^4+^.

### *In vivo* PET imaging of [^89^Zr(CP256)]^+^ and [^89^Zr(ox)_4_]^4–^

**Fig. 8:**
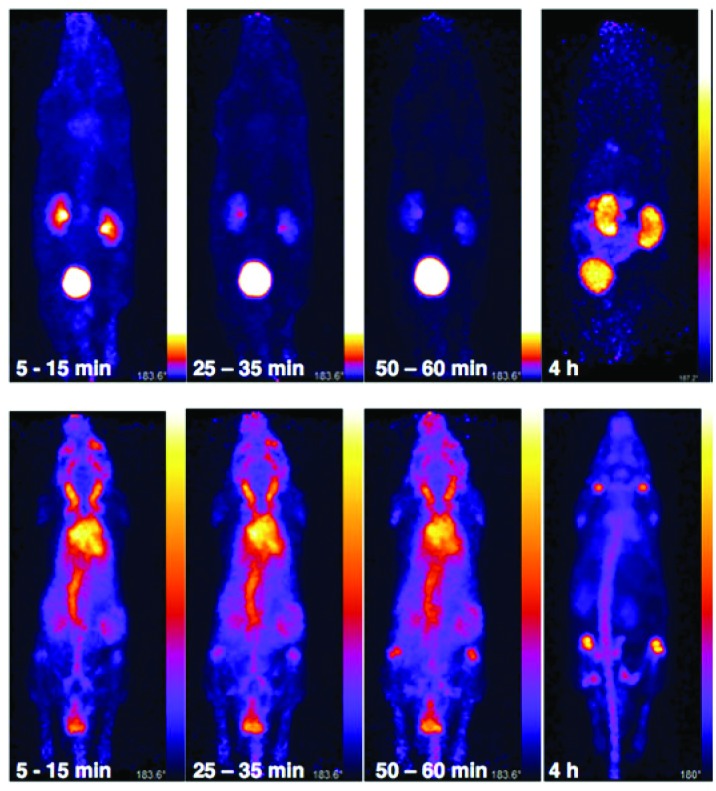
Whole body PET images of C57Bl/6j mice administered 10.3 MBq of [^89^Zr(CP256)]^+^ (mouse 3, top) and 9.3 MBq of [^89^Zr(ox)_4_]^4–^ (bottom). [^89^Zr(CP256)]^+^ clears rapidly from the blood pool *via* a renal pathway. At 10–15 min PI, 35% of injected dose was associated with the bladder, at 25–30 min PI, 55% of the injected dose was observed in the bladder and at 1 h PI, 74% activity of the injected does was associated with the bladder. At 4 h PI, approximately 4% of the original injected dose remained associated with the animal. In contrast, for the animal administered [^89^Zr(ox)_4_]^4–^, 98% of the injected dose remains 4 h PI, and is associated predominantly with the skeleton. Different scaling has been used for the top three images on the left, because non-bladder activity was so low that the kidneys could not be visualised without brightness/contrast enhancement.

The *in vivo* biodistribution and clearance pathway of [^89^Zr(CP256)]^+^ were assessed in C57B1/6j mice (*n* = 3) and compared to that of [^89^Zr(ox)_4_]^4–^ (*n* = 1) by PET (Fig. 8). The *in vivo* biodistribution of [^89^Zr(ox)_4_]^4–^ was consistent with previous studies utilising murine models,^15,42^ and so only a single animal was utilised in this study. We did not assess the *in vivo* biodistribution of [^89^Zr(DFO)]^+^ as this has been previously reported – the complex is excreted *via* a renal pathway within 20 min PI.^15^ Initially, [^89^Zr(ox)_4_]^4–^ circulated in the blood pool. By 15 min post-injection (PI), some ^89^Zr activity had accumulated at bones and joints (see knee joint in Fig. 8) and this continued to increase over time. Significant activity was associated with the blood pool during the first hour PI. By 4 h PI, ^89^Zr had significantly accumulated in bones and joints, although some activity was still observed in the blood pool with the animal retaining approximately 98% of the original injected dose (decay corrected, calculated from PET coincidence rates). In marked contrast, [^89^Zr(CP256)]^+^ cleared from the blood pool rapidly *via* a renal pathway (Fig. 8 and S7†). For the three animals used in this study, at 15 min PI, 15–35% of injected dose was associated with the bladder, and at 60 min PI, the majority of the injected activity was associated with the bladder (55–75% of the injected dose, Fig. 8 and S7†), although the kidneys (1.5–10% injected dose) were also visible in the whole body scan. This is comparable to what has previously been reported for [^89^Zr(DFO)]^+^.^15^ After 4 h PI, the fraction of the original injected dose remaining associated with each animal was 7% for mouse 1, 8% for mouse 2 and 4% for mouse 3, enabling the kidneys and bladder to be visualised in all three cases (*e.g.* Fig. 8).

### Biodistribution and PET imaging studies of ^89^Zr-BFC-trastuzumab conjugates

The *in vivo* renal clearance of [^89^Zr(CP256)]^+^ was too rapid to assess its stability *in vivo*. Furthermore, previous reports document similar rapid excretion of the [^89^Zr(DFO)]^+^ complex.^15^ The *in vivo* biodistributions of ^89^Zr-YM103-trastuzumab and ^89^Zr-DFO-trastuzumab were assessed in six weeks-old male C57Bl/6j mice by *ex vivo* biodistribution and *in vivo* PET imaging studies (*n* = 3 for each time point). Dissociated ^89^Zr^4+^ is thought to accumulate in bone joints and skeleton.^12,15,17,43^ Additionally, mAbs such as trastuzumab typically have long circulation times in the absence of tissues that are positive for the target receptor (as in this case, in which animals were not bearing tumours). For the immunoconjugates studied here, *in vivo* stability was assessed by quantifying persistence of ^89^Zr activity in the blood pool compared to accumulation in bones and joints.

**Fig. 9:**
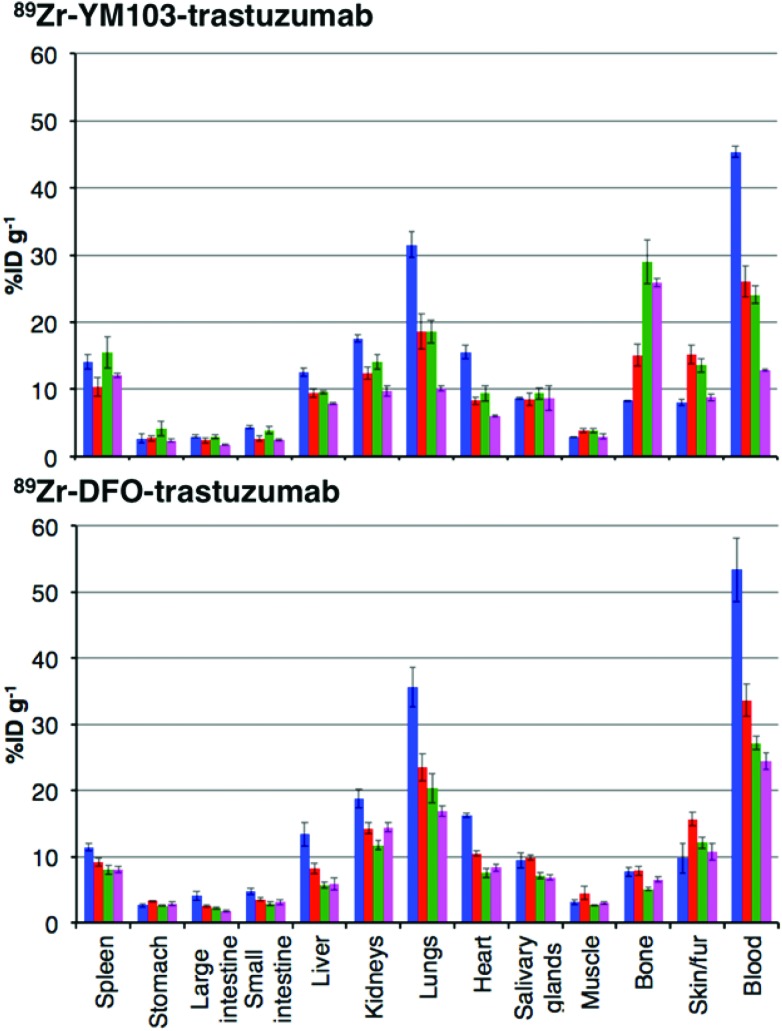
Biodistribution of ^89^Zr-labeled trastuzumab immunoconjugates in normal male C57Bl/6j mice at 6 h (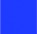), 1 day (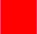), 3 days (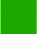), and 7 days (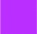) post-injection. Top: Biodistribution for animals administered ^89^Zr-YM103-trastuzumab; Bottom: Biodistribution for animals administered ^89^Zr-DFO-trastuzumab. Error bars correspond to standard error of the mean, (*n* = 3 for each timepoint). Notably, animals administered ^89^Zr-YM103-trastuzumab demonstrated significantly higher bone uptake and lower blood pool retention compared to animals administered ^89^Zr-DFO-trastuzumab over the course of the experiment. This is indicative of the lower *in vivo* stability of the ^89^Zr-chelate in ^89^Zr-YM103-trastuzumab compared to ^89^Zr-DFO-trastuzumab.

**Fig. 10:**
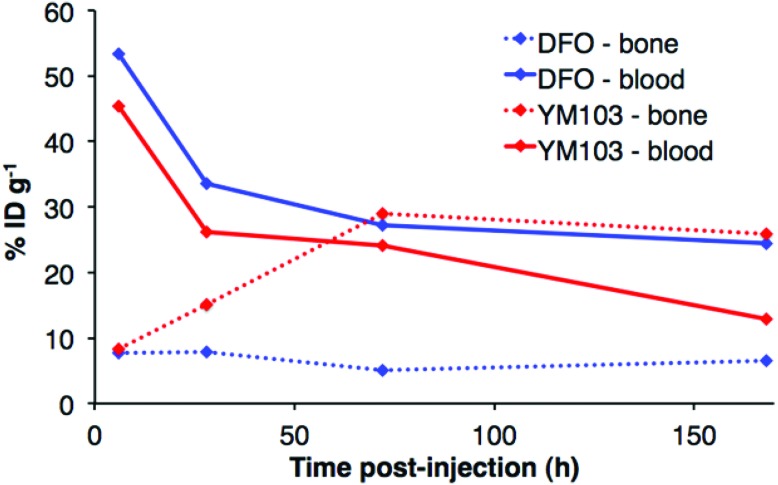
Comparison of ^89^Zr activity associated with blood pool and bone (%ID g^–1^) for animals administered ^89^Zr-YM103-trastuzumab and ^89^Zr-DFO-trastuzumab, determined by *ex vivo* biodistributions. At 7 days PI, bone uptake is higher than blood retention for animals administered ^89^Zr-YM103-trastuzumab, whereas for animals administered ^89^Zr-DFO-trastuzumab, activity in blood is higher than that associated with bone.

*Ex vivo* biodistribution data indicate that after 6 h PI, activity was associated with the blood pool for both immunoconjugates (^89^Zr-DFO-trastuzumab: 53.4 ± 4.8%ID g^–1^; ^89^Zr-YM103-trastuzumab: 45.4 ± 0.8%ID g^–1^), along with significant amounts of activity associated with the spleen, liver, kidneys, lungs and heart (Fig. 9). Bone uptake was moderate (^89^Zr-DFO-trastuzumab: 7.7 ± 0.7%ID g^–1^; ^89^Zr-YM103-trastuzumab: 8.3 ± 0.1%ID g^–1^). The biodistribution profiles of the two immunoconjugates were not significantly different at 6 h PI. At 1 day PI, significant amounts of activity were still associated with the blood pool for both ^89^Zr-DFO-trastuzumab (33.6 ± 2.4%ID g^–1^) and ^89^Zr-YM103-trastuzumab (26.1 ± 2.3%ID g^–1^). For ^89^Zr-DFO-trastuzumab, the relative bone-associated activity had not altered (7.8 ± 0.7%ID g^–1^), whereas for ^89^Zr-YM103-trastuzumab, relative bone-associated activity almost doubled (15.1 ± 1.6%ID g^–1^) compared to that observed at the 6 h PI time point (Fig. 10). Over the course of a week, relative bone-associated activity for animals injected with ^89^Zr-DFO-trastuzumab did not increase (5.1 ± 0.2%ID g^–1^ at 3 days PI, and 6.5 ± 0.4%ID g^–1^ at 7 days PI) but bone-associated activity for animals injected with ^89^Zr-YM103-trastuzumab increased to 29.0 ± 3.3%ID g^–1^ at 3 days PI, and did not change substantially thereafter (25.9 ± 0.6%ID g^–1^ at 7 days PI). For ^89^Zr-DFO-trastuzumab, blood associated activity decreased to 27.2 ± 1.0%ID g^–1^ at 3 days PI and slightly further to 24.4 ± 1.3%ID g^–1^ at 7 days PI. For ^89^Zr-YM103-trastuzumab, blood associated activity decreased to 24.1 ± 1.3%ID g^–1^ at 3 days PI and then significantly further to 12.9 ± 0.2%ID g^–1^ at 7 days PI. At the conclusion of the experiment (7 days), the ratio of blood/bone activity (as a ratio of %ID g^–1^ values) was 3.7 for animals injected with ^89^Zr-DFO-trastuzumab, whereas for animals injected with ^89^Zr-YM103-trastuzumab, the ratio was 0.5. For both immunoconjugates, the high %ID g^–1^ in lung, and its clearance, parallels that in the blood pool suggesting that the lung radioactivity is largely confined to blood and in this respect is typical of radiolabelled antibodies showing delayed clearance from blood.

**Fig. 11:**
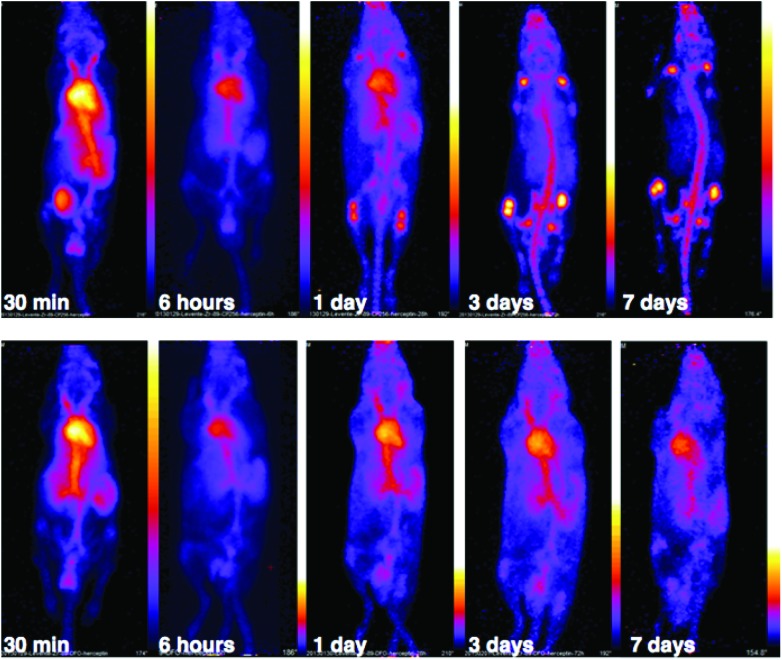
PET scans of C57Bl/6j mice administered 5–6 MBq of ^89^Zr-YM103-trastuzumab (top) and ^89^Zr-DFO-trastuzumab (bottom). PET images corroborate *ex vivo* biodistribution data, with the skeleton of the animal administered ^89^Zr-YM103-trastuzumab clearly visible from 3 days PI. This is in marked contrast to the animal administered ^89^Zr-DFO-trastuzumab, where the blood pool remains visible out to 7 days PI.

PET scans acquired at 0.5 h, 6 h, 1 day, 3 days and 7 days PI were consistent with biodistribution data for both ^89^Zr-labelled immunoconjugates (Fig. 11). PET images of animals imaged with ^89^Zr-labelled immunoconjugates exhibited predominantly blood pool associated activity at the 6 h PI time point. After this time point, the blood pool was still clearly visible out to 7 days PI for the animal administered ^89^Zr-DFO-trastuzumab, although some bone activity was observed – for example, the knee joints were visible from 1 day PI onwards. For the animal administered ^89^Zr-YM103-trastuzumab, the blood pool was visible by PET at 1 day PI, but from 3 days PI, activity was predominantly associated with the skeleton. Indeed, from 1 day PI, the knee joints of this animal were as prominent in the scan as the blood pool, and at 3 days PI, and even more so at 7 days PI, the vertebrae, skull and major joints of the animal were clearly distinguishable. The striking difference observed in the comparison of the ratios of blood : bone activity concentration (from *ex vivo* biodistribution data of the two ^89^Zr-labelled immunoconjugates at 7 days PI) was clearly discernable when qualitatively comparing the PET images for the same time point. Overall, the biodistribution and PET scanning data point to significantly lower *in vivo* stability of ^89^Zr-YM103-trastuzumab compared to ^89^Zr-DFO-trastuzumab with respect to dissociation of the ^89^Zr^4+^ metal complex.

## Discussion

When a methanolic or aqueous solution containing Zr^4+^ was added to a solution of H_3_CP256, a single signal, with a retention time of 8.03 min was observed in the UV-Vis chromatogram. The mass spectrum indicated that this compound corresponded to a Zr^4+^ complex of CP256, with a 1 : 1 stoichiometry. The mass spectrum did not reveal significant populations of dimeric or multimeric complexes, or 2 : 1 or 1 : 2 complexes, *etc.*, with intensities ≥ 5% (of normalised spectrum), indicating that the Zr^4+^ species in solution is monomeric with a stoichiometry of 1 : 1.

NMR samples prepared in the same way resulted in complex spectra with broad ^1^H NMR line shapes, presumably arising from fluxionality in the complex. The complexity of the ^1^H spectrum at 5 °C in the aromatic region and pyridinone CH_3_ region suggest that hydroxypyridinone groups are inequivalent and that multiple species exist in solution, that possibly interconvert *via* transient dissociation and re-coordination of hydroxypyridinone O ligand atoms. Indeed, comparatively sharper resonances at 5 °C with chemical shifts similar to that of the unbound ligand suggest that a population of compounds containing unbound hydroxypyridinone ligand arms could exist in solution. In light of the LCMS results that point to the presence of only a mononuclear 1 : 1 complex in solution, it is supposed that [Zr(CP256)]^+^ is highly fluxional with multiple interconverting isomeric species in solution. Dissociation and reassociation of solvent/water molecules to Zr^4+^ could also contribute to the observed fluxionality of the complex. The fluxional behaviour observed for [Zr(CP256)]^+^ on the ^1^H NMR timescale is likely indicative of the kinetic instability of the complex, and thus in the future ^1^H NMR spectroscopic studies could be useful for probing kinetic stability of diamagnetic complexes prior to *in vivo* testing.

In contrast, the [Zr(DFO)]^+^ complex gave rise to a spectrum with lineshapes that were sufficiently resolved to allow for assignment of NMR resonances in methanol-*d* ^4^. Observed broadening of resonances in spectra of [Zr(DFO)]^+^ at low temperatures was attributed to a component of fluxionality within the complex, where averaging of signals was observed for a proton that exchanges between different chemical environments. These different environments arise as a result of conversion between two or more thermodynamically and kinetically accessible species. Geminal protons (with the exception of protons labelled a–e (Fig. 3) are inequivalent and diastereotopic. It is possible that the fluxionality within the system is a result of dissociation and reassociation of water molecules, or conversion between enantiomers where geminal protons exchange environments. Neither process necessarily requires dissociation of Zr^4+^ from hydroxamate O ligands. Chemical exchange/dynamic processes in the system in deuterium oxide are slower than the corresponding processes in methanol-*d*_4_ at the same temperature (Fig. S2, ESI†), and at 25 °C in D_2_O, additional resonances are observed. Coalescence occurs at comparatively higher temperatures in the case of [Zr(DFO)]^+^ in deuterium oxide. The increased complexity at low temperature in deuterium oxide could be a result of separation of resonances for geminal protons that are involved in exchange processes.

Previous reports have described the presence of geometric isomers for octahedral metal complexes of DFO, arising from *cis*–*trans* isomers with respect to the coordinated O.^39^ These species result in multiple sets of signals in the ^13^C NMR spectrum in the ^13^C=O region, and it has been proposed that the rearrangement process would not necessitate a transition state involving dissociation of the ligand from the metal. In the case of Zr^4+^, which likely accommodates eight ligands,^15^ there was no description of this type of geometric isomerism in the DFT calculations. We only detect a single set of peaks in the ^13^C=O region of the ^13^C NMR spectrum. Indeed, if *cis*–*trans* isomers (arising from a change in coordination geometry of hydroxamate O ligands) interconvert in solution, the process cannot be detected by ^13^C NMR under the experimental conditions employed for this study, although mechanisms could be conceived by which such isomerism occurs, and such isomerism has been observed for an octadentate Zr^4+^ complex of four bidentate *N*-methyl acetohydroxamate ligands.^16^ Finally, on the basis of NMR data, primary amine coordination to Zr^4+^ cannot be ruled out, although in light of previously reported DFT calculations^15^ and the small differences in ^13^C chemical shift for methylene group a, this appears unlikely.

Competition studies demonstrated that H_3_CP256 was able to coordinate >85% of ^89^Zr^4+^ present in solution when H_3_DFO was present in a 10-fold excess, indicating that the conditional stability constant for [Zr(CP256)]^+^ is approximately two orders of magnitude greater than that of [Zr(DFO)]^+^.

When [^89^Zr(ox)_4_]^4–^ (at radiopharmaceutical concentrations) was reacted with a solution containing low concentrations of H_3_CP256 (10 μM or less), the radiochemical yield was less than 40% over the course of a two hour reaction. In contrast, solutions containing H_3_DFO under the same conditions (10 μM chelator) resulted in radiochemical yields over 80% at every time point. Furthermore, at micromolar concentrations (1 μM chelator), acceptable yields for H_3_DFO (80–85%) were achieved after two hours’ reaction time, in contrast to <30% for H_3_CP256 under the same conditions. In the context of radiosynthesis in a clinical radiopharmacy where very high specific activity may be required, a H_3_DFO conjugate will be more useful than the corresponding H_3_YM103 conjugate, despite the higher conditional stability constant of the latter complex.

Such results point to complex equilibria and kinetics, and demonstrate the limitations of *in vitro* studies in predicting *in vivo* behaviour. It is possible that a Zr^4+^ complex of CP256 is more thermodynamically stable than that of DFO, but that in an environment where ligand concentration is low (<100 μM) and mechanisms exist whereby dissociated Zr^4+^ is transported away from H_3_CP256 (such as some ITLC conditions employed in the concentration studies, or *in vivo*), the lability of [Zr(CP256)]^+^ results in dissociation and separation of Zr^4+^ from CP256.

The stability of [Zr(DFO)]^+^ and [Zr(CP256)]^+^ in the presence of excess Fe^3+^ indicated that [Zr(DFO)]^+^ is (kinetically, presumably) stable in the presence of excess Fe^3+^, but that CP256 partially dissociates from Zr^4+^ and coordinates to Fe^3+^. This behaviour is consistent with the greater lability of [Zr(CP256)]^+^ compared to [Zr(DFO)]^+^. The concentration of Fe^3+^ utilised in this experiment is not biologically relevant, but does allow for a qualitative comparison of the kinetic stabilities of [Zr(DFO)]^+^ and [Zr(CP256)]^+^. It is seemingly inconsistent that [^89^Zr(DFO)]^+^ is stable in the presence of Fe^3+^ but dissociates in the presence of 0.1 equivalent of H_3_CP256 relative to H_3_DFO. Zr^4+^ can accommodate up to eight ligands in its coordination sphere, and it is possible that the reaction mechanisms governing the kinetics of transchelation involve reaction intermediates or transition states where ^89^Zr^4+^ is coordinated to both DFO and CP256. In the case of the Fe^3+^ competition studies, the competing ligand is absent.

Under the same reaction conditions, the conjugation of H_3_YM103 to trastuzumab was less efficient than the conjugation of a maleimidopropionate-H_3_DFO derivative to trastuzumab. This reduced reactivity for H_3_YM103 is possibly a consequence of the increased steric bulk of the hydroxypyridinone rings compared to the linear H_3_DFO chain, resulting in a decrease in the number of chelators that can be accommodated at reduced cysteine side chains in close proximity to one another, or consequently a decrease in the rate of reaction at these cysteines.

The maximum specific activity obtained for ^89^Zr-YM103-trastuzumab was just over half that achieved for ^89^Zr-DFO-trastuzumab. Lower concentrations of YM103-trastuzumab (0.5 fold lower) resulted in lower radiochemical yields of approximately 76%. This is not surprising in light of the concentration studies that demonstrated that H_3_DFO coordinates ^89^Zr^4+^ to give high radiochemical yields (>80%) at lower concentrations of chelator. It is also consistent with the observation that there were less H_3_YM103 chelators present per molecule of antibody compared to H_3_DFO. Higher specific activities could potentially be achieved at the expense of radiochemical yield necessitating the inclusion of a purification step.

Serum stability studies conducted over 7 days indicated that <5% ^89^Zr^4+^ dissociated from YM103-trastuzumab or DFO-trastuzumab in serum. However, results from the biodistribution data and PET images indicate that the [^89^Zr(YM103)]^+^ group is not sufficiently stable *in vivo*, and after one day, significant amounts of ^89^Zr had dissociated from the tris(hydroxypyridinone) chelator and accumulated at the skeleton of the animal. At 3 days PI, and even more so at 7 days PI, the PET image resembles that of the animal administered [^89^Zr(ox)_4_]^4–^ at the 24 h PI time point. For the animals administered ^89^Zr-DFO-trastuzumab, observed bone uptake was consistent with previous reports of ^89^Zr-labelled DFO-protein conjugates. These reports have attributed this bone uptake to either release of ^89^Zr^4+^ from the chelator, or metabolism of the labelled immunoconjugate resulting in accumulation of ^89^Zr-metabolites in the bone marrow.^12,15,17^ In light of the biodistribution profile we observe for animals administered ^89^Zr-YM103-trastuzumab, we favour interpreting this bone uptake as deposition of released ^89^Zr^4+^ in bone mineral, rather than accumulation of metabolised fragments of ^89^Zr-DFO-trastuzumab. Consistent with this are the reports of significantly lower bone uptake at comparable time points (3 days and 7 days) for mice administered ^111^In-labelled immunoconjugates.^3,22,44,45^

Geometric rigidity and kinetic inertness of radiolabelled complexes is demonstrably important for *in vivo* stability for other radiometallic imaging agents,^7,46,47^ and in the case of ^89^Zr^4+^, this is also critical. Despite its higher thermodynamic stability at neutral pH compared to ^89^Zr-DFO, the lability of the [Zr(YM103)]^+^ complex *in vivo* leads to either competition from endogenous ligands (either serum proteins^43^ such as apo- or holo-transferrin^46^ or inorganic mineral anions such as phosphate in hydroxyapatite^42^) for ^89^Zr^4+^ binding; or competition from endogenous metal ions, such as Fe^3+^,^31,33^ for hydroxypyridinone coordination.

## Concluding remarks

Lability in an octadentate Zr^4+^ complex of four *N*-methyl acetohydroxamate (Me-AHA) ligands, [Zr(Me-AHA)_4_] has been previously observed and attempts at radiolabelling Me-AHA with ^89^Zr^4+^ to give [^89^Zr(Me-AHA)_4_] resulted in radiochemical yields of only 20%, as opposed to H_3_DFO that gave >99%.^16^ These results indicate that the prearrangement of hydroxamate groups in a multidentate ligand is important for coordination of ^89^Zr^4+^. It is possible that the topology of the ligand is an important factor in determining kinetic stability. The linear arrangement of the three hydroxamate groups in H_3_DFO might contribute to the inertness of [Zr(DFO)]^+^. The tripodal arrangement of hydroxypyridinone groups in H_3_CP256 and H_3_YM103 might not be optimal for high kinetic stability in [Zr(CP256)]^+^ and Zr(YM103)]^+^. It would be instructive to compare the lability of [^89^Zr(DFO)]^+^ with that of ^89^Zr^4+^ complexes of existing tripodal tris(hydroxamate) and cryptate tris(hydroxamate) ligands.^48^ However, in light of the structure of [Zr(Me-AHA)_4_],^16^ it is likely that the incorporation of four rather than three bidentate chelators into a suitable topology will be required to improve on the Zr-chelating properties of DFO. Indeed, recently reported stability constant data indicates a preference for 1 : 4 complexes for the Me-AHA complex.^16^ We, like others,^16,27^ predict that replacing a hexadentate ligand with an octadentate ligand containing eight coordinating O atoms could increase the specific activity, inertness and complex stability of a ^89^Zr-labeled immunoconjugate, reducing the bone uptake observed in small animal rodents that is observed for H_3_DFO-immunoconjugates.

Determination of stability constants of complexes of hydroxypyridinone ligands and Zr^4+^ was beyond the scope of this present work, and so we cannot comment on the quantitative thermodynamics of hydroxypyridinone ligands *vs.* hydroxamate ligands. Nevertheless the competition studies suggest that the conditional stability constant for [Zr(CP256)]^+^ under the conditions of our experiments are around two orders of magnitude higher than [Zr(DFO)]^+^. Zr^4+^ can achieve coordination geometries that accommodate up to eight ligands, and it is possible the transchelation of ^89^Zr^4+^ from DFO to CP256 (and *vice versa*) involves a ternary intermediate or transition state where Zr^4+^ bridges both ligands. Such a mechanism allows for rapid release of ^89^Zr^4+^ from DFO. However, in the presence of Fe^3+^ with no competing ligands, [^89^Zr(DFO)]^+^ is stable within the timeframe of the experiment. On the other hand, the hydroxypyridinone groups of [^89/nat^Zr(CP256)]^+^ are labile, and NMR evidence indicates that it is likely that populations exist where hydroxypyridinone groups are not coordinated to Zr^4+^. This enables effective Fe^3+^ competition, and it is likely that in the case of transmetallation of CP256 from ^89^Zr^4+^ to Fe^3+^, reaction intermediates involve a CP256 ligand that bridges both Zr^4+^ and Fe^3+^ metal ions.

Whilst the competition studies demonstrated that thermodynamically, at radiochemical concentrations, ^89^Zr^4+^ preferentially coordinates to H_3_CP256 over H_3_DFO, the kinetic lability of the [Zr(YM103)]^+^/[Zr(CP256)]^+^ complex is considerably greater than that of [Zr(DFO)]^+^, giving rise to both significant broadening of signal resonances on the NMR timescale and ultimately, substantially lower *in vivo* stability. Metal complex intermediates where a ligand arm has dissociated from Zr^4+^, allowing endogenous competing ligands to complex Zr^4+^ (either serum proteins^43^ such as apo- or holo-transferrin^41^ or inorganic mineral anions such as phosphate in hydroxyapatite^42^), or endogenous metal ions, such as Fe^3+^,^29,31^ to coordinate to the dissociated ligand atoms, are likely to play a role in the *in vivo* dissociation and transchelation/transmetallation pathways. Hence, transchelation rates and mechanisms in one context do not predict those in another – *e.g.* Zr^4+^ transchelates rapidly from DFO to CP256, yet [Zr(DFO)]^+^ is more inert than [Zr(CP256)]^+^ towards competition with Fe^3+^. While the mechanisms of release or transchelation of Zr by the chelating agents are complex and not properly understood, it is clear that in practical terms, bifunctional chelator derivatives of H_3_DFO are preferable to bifunctional chelator derivatives of tris(hydroxypyridinone)/H_3_CP256 for ^89^Zr PET imaging with immunoconjugates where prolonged *in vivo* stability is required.

## Experimental

### Materials and instrumentation

Chemicals and reagents were obtained from Sigma-Aldrich (Dorset, UK) unless otherwise specified. The highest available purity (lowest metal ion-containing) chemicals were used. Sterile water for injection, used to prepare buffers, was obtained from Baxter Healthcare (Newbury, UK). G-25 Illustra NAP-5 size exclusion columns were purchased from Fisher Scientific Ltd (Leicestershire, UK) and washed with 0.1 M ammonium acetate solution, pH 6. Trastuzumab (MabThera, Roche) was obtained as a 10 mg mL^–1^ solution from the Pharmacy Department at Guy's and St. Thomas’ NHS Trust, London. Fresh female O^+^ type human serum was obtained from a healthy volunteer. High-performance liquid chromatography (HPLC) analysis was carried out using an Agilent 1200 LC system with in-line UV and gamma detection (Flow-Count, LabLogic). NMR spectra were acquired on a Bruker Avance 400 spectrometer (Bruker UK Limited, Coventry, UK) equipped with a 5 mm QNP probe at 400.13 MHz for ^1^H NMR spectra (using a zg30 pulse program) and 100.6 MHz for ^13^C NMR spectra (using a zgpg30 pulse program), or in the case of low temperature variable temperature experiments, a Bruker Avance 500 spectrometer with a triple resonance cryoprobe with z-gradients at 500 MHz for ^1^H NMR spectra (using a zg30 pulse program), or in the case of high temperature variable temperature experiments in deuterium oxide, a Bruker Avance 400 spectrometer with a PH BBO probe at 400.13 MHz for ^1^H NMR spectra (using a zg30 pulse program). Spectra were referenced to residual solvent signals, or in the case of deuterium oxide, an acetone reference. Mass spectra were recorded in the positive ion mode on an Agilent 6520 Q-TOF LC/MS mass spectrometer coupled to an Agilent 1200 LC system (Agilent, Palo Alto, CA). Data were acquired and reference mass-corrected *via* a dual-spray electrospray ionisation source, using the factory-defined calibration procedure. Analytical reverse phase LCMS and radio-LCMS traces were acquired using an Eclipse XDB-C18 column (4.6 × 150 mm, 5 μm) with a 0.5 mL min^–1^ flow rate and UV spectroscopic detection at either 214 nm, 256 nm or 270 nm. The LCMS was coupled to a LabLogic Flow-Count detector with a sodium iodide probe (B-FC-3200) to give a triple-readout chromatogram (UV-vis, radioactivity and total ion count). Instant thin layer chromatography strips (ITLC-SG) were obtained from Varian Medical Systems UK, Ltd. (Crawley, UK), and ITLC strips were visualised using a Perkin Elmer Storage Phosphor System (Cyclone). Analytical reverse phase HPLC and radio-HPLC traces were acquired using an Agilent 1200 LC system and an Agilent Zorbax Eclipse XDB-C18 column (4.6 × 150 mm, 5 μm) with a 1 mL min^–1^ flow rate and UV spectroscopic detection at either 214 nm, 256 nm or 270 nm. Analytical size exclusion radio-HPLC traces were acquired using an Agilent 1200 Series HPLC system and a Phenomenex Biosep 2000 (300 × 7.8 mm) size exclusion column with ammonium acetate (0.1 M) mobile phase and, unless otherwise specified, sodium ethylenediamine tetraacetate (2 mM). The radio-HPLC was coupled to a LabLogic Flow-Count detector with a sodium iodide probe (B-FC-3200). Aliquots (10–200 μL) of each radiolabelled sample were injected onto the column, using a flow rate of 1 mL min^–1^.

Zirconium-89: no-carrier-added zirconium-89 (radionuclidic purity >99.9%) produced at the BV Cyclotron, Amsterdam, was purchased from Perkin Elmer. Solutions containing H_4_[^89^Zr(ox)_4_] (ox = oxalate) in aqueous oxalic acid (1 M) were titrated with sodium carbonate (1 M) until pH 6–7 (measured by pH strips) was obtained.

Multidentate chelators and bifunctional derivatives: the tris(hydroxypyridinone) derivatives, H_3_CP256 and H_3_YM103, were prepared according to previously reported procedures.^33,38^ Desferrioxamine (mesylate salt) was purchased from Sigma-Aldrich and used as received. Maleimidopropionate-desferrioxamine was synthesised according to a previously reported procedure,^12^ using 3-(maleimido)propionic acid (*N*-hydroxysuccinimide ester) (purchased from Alfa Aesar, Ward Hill, Massachusetts). ESI-MS: [C_32_H_53_N_7_O_11_]^+^, *m*/*z* = 712.39 (experimental), 712.39 (calculated). ^1^H NMR DMSO-*d*_6_ *δ* 1.21, m, 6H; 1.36, m, 6H; 1.49, m, 6H; 1.96, s, 3H; 2.26, t, ^3^ *J* 7.32, 4H; 2.30, t, ^3^ *J* 7.26, 2H; 2.57, t, ^3^ *J* 7.13, 4H; 2.98, m, 6H; 3.44 (integration and splitting obscured by HDO/H_2_O signal), presumably 6H; 3.59, t, ^3^ *J* 7.26, 2H; 6.99, s, 2(CH); 7.79, t, ^3^ *J* 5.07, 2(NH); 7.92, t, ^3^ *J* 5.49, 1(NH); 9.65, broad s, 2(OH); 9.71, broad s, 1(OH). ^13^C NMR DMSO-*d*_6_ *δ* 20.4, 23.6, 26.1, 27.7, 28.7, 28.9, 30.0, 34.2, 34.3, 38.4, 38.5, 46.9, 47.2, 134.6, 169.4, 170.3, 170.9, 171.5, 172.1.

### *In situ* preparation of Zr^4+^ complexes of H_3_CP256 and H_3_DFO for NMR spectroscopy

[Zr(acac)_4_] (zirconium acetylacetonate) (1–1.3 equivalents) in methanol-*d*_4_) was added to H_3_CP256 in methanol-*d*_4_. Similarly, [Zr(DFO)]^+^ was prepared by addition of [Zr(acac)_4_] (1–1.3 equivalents) in methanol-*d*_4_ to H_3_DFO in methanol-*d*_4_. Samples were also prepared using deuterium oxide in place of methanol-*d*_4_. Spectroscopic data are included in Fig. 1–3, Table 1 and ESI.†

### Immunoconjugate preparation

A solution of sodium ethylenediaminetetracetate (3 μL, 50 mM, 10 equivalents) was added to a solution of trastuzumab (100 μL, 20 mg mL^–1^ in 0.9% aqueous NaCl) to chelate any adventitious metal ions present. A solution of tris(2-carboxyethyl)phosphine hydrochloride (3 μL, 50 mM, 10 equivalents) was added to the solution of trastuzumab and the mixture heated at 37 °C for 30 min. The BFC derivatives (20 equivalents) were added to these reaction solutions (H_3_YM103 – 0.25 mg in 2.5 μL DMSO; maleimidopropionate-H_3_DFO – 0.20 mg in 4 μL DMSO), and the mixtures heated at 37 °C for 30 min. The reaction solutions were each loaded onto an Illustra NAP-5 size exclusion column that had been rinsed with aqueous ammonium acetate solution (0.1 M). The column was eluted with ammonium acetate solution (0.1 M) and each fraction (0.5 mL) was analysed by size exclusion HPLC (*λ* = 280 nm). In both cases, the second fraction contained approximately half of the total amount of immunoconjugate and subsequent size exclusion chromatography after radiolabelling experiments with this fraction demonstrated that negligible amounts of unreacted bifunctional chelator were present. In contrast, radiolabelled products in fraction three included immunoconjugate and low molecular mass compounds, presumably unreacted bifunctional chelator that is available to react with [^89^Zr(ox)_4_]^4–^. Fraction two was used exclusively for all further experiments that utilised H_3_DFO-trastuzumab and H_3_YM103-trastuzumab. Mass spectra were acquired using a 350 V fragmentor voltage and a 1500 V capillary voltage, and were deconvoluted using Agilent MassHunter Qualitative Analysis software, using factory settings for deconvolution with maximum entropy.

### Radiolabelling

LCMS studies: a solution containing [^89^Zr(ox)_4_]^4–^ (500 kBq, 2.5 μL) was added to a solution of H_3_CP256 (20 μL, 1 mM in 0.1 M ammonium acetate solution), and incubated at room temperature for 10–15 min. After this time, an aliquot was analysed by LCMS (flow rate: 0.5 mL min^–1^; gradient mobile phase: 100%/0% solvent A/B at 0 min to 0%/100% solvent A/B at 20 min; solvent A: 0.1% formic acid in water; solvent B: 0.1% formic acid in acetonitrile), and the chromatogram compared with that of [^nat^Zr(CP256)]^+^, prepared by addition of [Zr(acac)_4_] (1–2 equiv.) to a solution of H_3_CP256 (20 μL, 1 mM). LCMS [^nat^Zr(CP256)]^+^: retention time: 8.00 min; [Zr(C_36_H_46_N_7_O_10_)]^+^: found 413.61 ((M + H)^2+^) and 826.22, (M^+^), calculated 413.62 and 826.24, respectively; LCMS [^89^Zr(CP256)]^+^: UV-Vis chromatogram, *λ*_254_ retention time: 8.01 min, radiochromatogram retention time: 8.61 min. LCMS [^89^Zr(ox)]^4–^: retention time: 3.13 min. Difference in retention times for the same species between UV and radio-scintillation HPLC chromatograms is a result of the configuration of the detectors in series.

Concentration dependence of labelling efficiency: a solution containing [^89^Zr(ox)_4_]^4–^ (200 kBq, 10 μL, adjusted to pH 6–7 with sodium carbonate as described above) was added to solutions of H_3_CP256 (each 10 μL, [H_3_CP256] = 10 mM, 1 mM, 100 μM, 10 μM, 1 μM and 100 nM in 0.1 M ammonium acetate solution) and the solutions were left to react at ambient temperature. Aliquots were analysed at 10, 30, 60 and 120 min using ITLC-SG strips using an aqueous citrate buffer (0.1 M, pH 5.5) mobile phase. The experiment was undertaken in triplicate, and each measurement was acquired in duplicate. The same reactions were also conducted using H_3_DFO in place of H_3_CP256. Activity was visualised by exposure (1–3 min) to a Perkin Elmer super resolution Storage Phosphor Screen, which was then imaged using a Perkin Elmer Cyclone Plus Storage Phosphor System at an image resolution of 300 dpi. To quantify activity, regions of interest (ROI) were selected and the gross digital light units (DLU) of each ROI summed. A background constant (DLU mm^–2^) was determined from an appropriate background area of each image, and subtracted from each ROI. Radiochemical yield was calculated as a fraction of the net DLU of the ROI corresponding to either the CP256 or DFO complex relative to the sum of DLU for all ROI for the specific ITLC strip. [^89^Zr(CP256)]^+^: *R*_f_ = 0, [^89^Zr(DFO)]^+^: *R*_f_ = 0–0.5, [^89^Zr(ox)_4_]^4–^: *R*_f_ > 0.8.

Competition studies: a solution containing [^89^Zr(ox)_4_]^4–^ (400 kBq, 2 μL) was added to solutions of H_3_CP256 (1 mM, 10 μL in 0.1 M ammonium acetate) and left to react for 10 min. After this time, aliquots containing H_3_DFO (1 mM, 10 mM or 100 mM, each 10 μL in 0.1 M ammonium acetate) were added. Each reaction was analysed using reverse phase C18 HPLC at 1 h and 12 h (flow rate: 1 mL min^–1^; gradient mobile phase: 100%/0% solvent A/B at 0 min to 70%/30% solvent A/B at 12 min; solvent A: 0.1% trifluoroacetic acid in water; solvent B: 0.1% trifluoroacetic acid in acetonitrile). In a similar experiment, [^89^Zr(ox)_4_]^4–^ (400 kBq, 2 μL) was added to solutions of H_3_DFO (1 mM, 10 μL in 0.1 M ammonium acetate) and left to react for 10 min. After this time, aliquots containing H_3_CP256 (1 mM, 10 mM or 100 mM, each 10 μL in 0.1 M ammonium acetate) were added, and the reactions were also analyzed by C18 HPLC at 1 h and 12 h. Additionally, a solution containing [^89^Zr(ox)_4_]^4–^ (400 kBq, 2 μL) was added to solutions of H_3_DFO at higher concentrations (100 mM and 10 mM, both 10 μL in 0.1 M ammonium acetate) and left to react for 10 min. After this time, aliquots containing H_3_CP256 (1 mM, 10 μL in 0.1 M ammonium acetate) were added to each solution and the reactions analysed by C18 HPLC after 1.5 h reaction time. Retention times for detection with a sodium iodide radio-HPLC probe: [Zr(CP256)]^+^: 8.52 min; [Zr(DFO)]^+^: 8.03 min.

Fe^3+^ competition studies: a solution containing [^89^Zr(ox)_4_]^4–^ (250 kBq, 0.5 μL) was added to solutions of H_3_CP256 (in 0.1 M ammonium acetate) and left to react for 10 min. After this time, a solution of FeCl_3_ (10 mM, 1 μL) was added to each solution of [^89^Zr(CP256)]^+^ (final [Fe^3+^] = 1 mM; final [H_3_CP256] = 1 mM or 100 μM; volume = 11 μL in 0.1 M ammonium acetate). After 20 min, aliquots of each reaction were analysed by ITLC as described above. Alongside these solutions, separate reaction solutions that did not contain Fe^3+^ were also prepared, so as to be able to compare the amount of intact [^89^Zr(CP256)]^+^ in Fe^3+^ solutions with radiochemical yield in the absence of Fe^3+^. The same reactions were also conducted using H_3_DFO in place of H_3_CP256. All reactions were undertaken in triplicate.

Conjugate labelling: ^89^Zr-DFO-trastuzumab for PET imaging: a solution containing [^89^Zr(ox)_4_]^4–^ (12.5 MBq, 21 μL) was added to a solution containing H_3_DFO-trastuzumab (125 μL, 1.1 mg mL^–1^, 138 μg). After incubation at ambient temperature for 5–10 min, size exclusion HPLC analysis demonstrated that the ^89^Zr-DFO-trastuzumab was present with radiochemical yield and purity of 98.3% and a specific activity of 91 MBq mg^–1^ of immunoconjugate (retention time for detection with a sodium iodide radio-HPLC probe: 7.12 min). ^89^Zr-YM103-trastuzumab for PET imaging: a solution containing [^89^Zr(ox)_4_]^4–^ (12.5 MBq, 21 μL) was added to a solution containing H_3_YM103-trastuzumab (260 μL, 0.8 mg mL^–1^, 208 μg). After incubation at ambient temperature for 5–10 min, size exclusion HPLC analysis demonstrated that the ^89^Zr-YM103-trastuzumab was present with a radiochemical yield of 98.7% and a specific activity of 55 MBq mg^–1^ of immunoconjugate (retention time for detection with a sodium iodide radio-HPLC probe: 7.12 min). Both solutions of ^89^Zr-labeled immunoconjugate were diluted to 300 μL with 0.1 M ammonium acetate for duplicate injections.

For dose injections into animals for *ex vivo* biodistribution studies, lower amounts of activity but a comparable amount of antibody were required per animal, relative to dose injections for animals used for acquisitions of PET images. As a result, the specific activities of solutions of ^89^Zr-labelled immunoconjugates for *ex vivo* biodistributions were necessarily lower than those prepared for PET imaging. ^89^Zr-DFO-trastuzumab for *ex vivo* biodistribution studies: a solution containing [^89^Zr(ox)_4_]^4–^ (7.4 MBq, 14 μL) was added to a solution containing H_3_DFO-trastuzumab (875 μL, 1.2 mg mL^–1^, 1.05 mg). After incubation at ambient temperature for 5–10 min, size exclusion HPLC analysis demonstrated that the ^89^Zr-DFO-trastuzumab was present with a radiochemical yield and purity of >99% and a specific activity of 7.05 MBq mg^–1^ of immunoconjugate. ^89^Zr-YM103-trastuzumab for *ex vivo* biodistribution studies: a solution containing [^89^Zr(ox)_4_]^4–^ (7.4 MBq, 14 μL) was added to a solution containing H_3_YM103-trastuzumab (600 μL, 1.7 mg mL^–1^, 1.02 mg). After incubation at ambient temperature for 5–10 min, size exclusion HPLC analysis demonstrated that the ^89^Zr-YM103-trastuzumab was present with a radiochemical yield and purity of >99% and a specific activity of 7.3 MBq mg^–1^ of immunoconjugate. These ^89^Zr-immunoconjugate solutions were both diluted to 1.3 mL to provide 12 injections (100 μL each) for biodistribution studies.

Serum stability studies: ^89^Zr-DFO-trastuzumab and ^89^Zr-YM103-trastuzumab were radiolabelled as described above to achieve specific activities of 14 MBq mg^–1^. An aliquot of each immunoconjugate (30 μL, 0.11 mg of immunoconjugate, 1.5 MBq) was incubated with fresh serum (200 μL O^+^ fresh human serum from a healthy female volunteer) for 24, 48 or 168 h, after which it was analysed using size exclusion HPLC.

### Determination of *K*_d_ values for ^89^Zr-YM103-trastuzumab and ^89^Zr-DFO-trastuzumab in a competitive binding assay

HCC1954 cells were grown in T175 flasks in culture medium. Cells were aspirated and washed with phosphate buffered saline (PBS), treated with trypsin to detach the cells from the flask and washed again with PBS. The cells were resuspended in Hank's buffered saline solution (HBSS) with 0.2% bovine serum albumin (BSA), aliquoted to give 2 × 10^5^ cells per tube, centrifuged at 1000 rpm for 3 min and aspirated. Solutions containing ^89^Zr-YM103-trastuzumab (1 nM) and trastuzumab (1–2000 nM) in HBSS with 0.2% BSA (1 mL) were incubated with the cells on ice for 1 h in triplicate. The cells were washed 3 times by centrifuging at 1000 rpm for 3 min, aspirating the supernatant and washing with ice-cold HBSS (1 mL). After this, radioactivity associated with cell pellets was counted using a Wallac 1282 Compugamma Universal Gamma Counter. A one site total binding fit using Prism 5.04 for Windows was used to fit the curves and determine the dissociation constants. The above procedure was also used to determine the dissociation constant for ^89^Zr-DFO-trastuzumab.

### *In vivo* biodistribution and PET imaging studies

*In vivo* PET imaging of [^89^Zr(CP256)]^+^ and [^89^Zr(ox)_4_]^4–^: six weeks-old male C57Bl/6j mice (*n* = 3) were purchased from Harlan UK and left to acclimatise for five days before use with *ad libitum* access to water and diet. PET images were acquired in a nanoScan® PC *in vivo* pre-clinical PET/CT imager (Mediso, Hungary). For PET imaging a mouse was anesthetised by inhalation of a 3% isoflurane–oxygen mixture and placed on the scanner bed; general anaesthesia was maintained with a 1.5–2.5% isoflurane–oxygen mixture. One lateral tail vein was cannulated with a 27 ga cannula. A PET scan was started and approximately one min later the mouse was injected with 10–12 MBq of [^89^Zr(CP256)]^+^ ([H_3_CP256] = 7 mM) in 100–200 μL ammonium acetate solution (0.1 M) *via* the cannulated vein. The mouse was scanned for a further 60 min and allowed to recover from anaesthesia. 30 min whole body PET scans were also acquired at 4 h post-injection (PI) under isoflurane anaesthesia. After the final PET scan the mouse was killed by cervical dislocation. This protocol was also used to image the tissue distribution of [^89^Zr(ox)_4_]^4–^; one mouse was injected with 9.3 MBq of [^89^Zr(ox)_4_]^4–^ in 200 μL ammonium acetate solution (0.1 M) *via* a cannulated tail vein and imaged as described above.

*In vivo* biodistribution and PET imaging of ^89^Zr-immunoconjugates: six weeks-old male C57Bl/6j mice were purchased from Harlan UK and left to acclimatise for five days before use with *ad libitum* access to water and diet. Twelve mice were injected intravenously (tail vein) with 500 kBq (80 μg) of ^89^Zr-DFO-trastuzumab and twelve with 500 kBq (130 μg) of ^89^Zr-YM103-trastuzumab. The biodistribution of the radiolabelled antibody conjugates was assessed at 6 h, 1 day, 3 days and 7 days PI (*n* = 3 per time point) as follows. At each time point mice were culled by CO_2_ asphyxiation. Blood samples (0.3–0.8 mL) were collected by cardiac puncture. Mice were dissected and the major thoraco-abdominal organs, salivary glands, thigh muscle and femora collected for *ex vivo* tissue counting. Tissue samples were washed in saline to remove excess blood then dried on highly absorbent tissue paper and placed in scintillation vials. Samples were weighed and activities measured in a Wallac 1282 Compugamma Universal Gamma Counter. Radiotracer accumulation in each tissue was calculated as the percentage of injected dose and normalised for the weight of the sample (%ID g^–1^). For imaging studies two mice were injected intravenously with 5–6 MBq (60–70 μg) of ^89^Zr-DFO-trastuzumab and two mice were injected with 5–6 MBq (120–130 μg) of ^89^Zr-YM103-trastuzumab. Whole-body PET scans were acquired in a nanoPET scanner at 0.5 h, 6 h, 1 day, 3 days and 7 days PI. Mice were culled by cervical dislocation at the end of the study.

All studies were approved by the institutional committee and conducted in strict compliance with Home Office (UK) guidelines on animal experimentation and the corresponding personal and project licenses.
