# Supplementary material for: Tripodal tris(hydroxypyridinone) ligands for immunoconjugate PET imaging with 89Zr4+: comparison with desferrioxamine-B
Source: Dalton Trans. 2014 Oct 29;44(11):4884–900. doi: 10.1039/c4dt02978j (PMC4357251; doi:10.1039/c4dt02978j)
Supplement: Supplementary file 1 [file DT-044-C4DT02978J-s001.pdf]

Electronic Supplementary Material (ESI) for Dalton Transactions.
This journal is © The Royal Society of Chemistry 2014

Tripodal tris(hydroxypyridinone) ligands for immunoconjugate PET imaging with $^{89}Zr^{4+}$: comparison with desferrioxamine-B

Ma, Michelle T.[1]; Meszaros, Levente K.[1]; Paterson, Brett M.[2]; Berry, David J.[1]; Cooper, Maggie S.[1]; Ma, Yongmin[3]; Hider, Robert C.[4]; Blower, Philip J.[1,5]

[1]King's College London, Division of Imaging Sciences and Biomedical Engineering, 4th Floor Lambeth Wing, St Thomas' Hospital, London SE1 7EH, UK; [2]School of Chemistry and Bio21 Molecular Science and Biotechnology Institute, The University of Melbourne, Parkville, Victoria 3052, Australia; College of Pharmaceutical Science, Zhejiang Chinese Medical University, Hangzhou, People's Republic of China, 310053; [4]King's College London, Institute of Pharmaceutical Science, Franklin Wilkins Building, Stamford St, London SE1 9NH, UK; [5]King's College London, Division of Chemistry, Britannia House, 7 Trinity St, London SE1 1DB

Corresponding authors: michelle.ma@kcl.ac.uk, philip.blower@kcl.ac.uk

Figure S1. ^{1}H NMR spectrum of $[Zr(CP256)]^+$ in deuterium oxide at 25 °C **Page 2**

Figure S2. Variable temperature ^{1}H NMR spectra of $[Zr(DFO)]^+$ in deuterium oxide **Page 2**

Figure S3. ^{13}C NMR spectra of H_3DFO and $[Zr(DFO)]^+$ in deuterium oxide at 25 °C **Page 3**

Figure S4. Reverse phase radiochromatograms of H_3DFO/H_3CP256 competition studies for $^{89}Zr^{4+}$ **Page 4**

Figure S5. Phosphoimages of ITLC plates **Page 5**

Figure S6. Size exclusion HPLC radiochromatograms of trastuzumab conjugates and serum stability studies **Page 6**

Figure S7. Percentage injected dose of $[^{89}Zr(CP256)]^+$ in kidneys and bladders of three normal male C57Bl/6j mice at 5 – 60 min post-injection **Page 6**

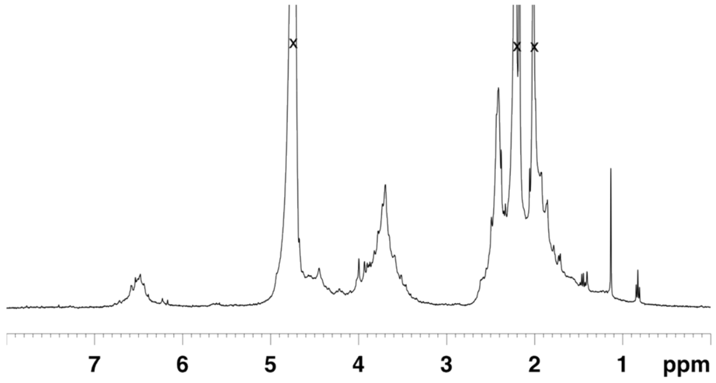

Figure S1. ^1H NMR spectrum of $[Zr(CP256)]^+$ in deuterium oxide at 25 °C. HDO, acetone, excess zirconium acetylacetonate and unbound acetylacetone marked (×).

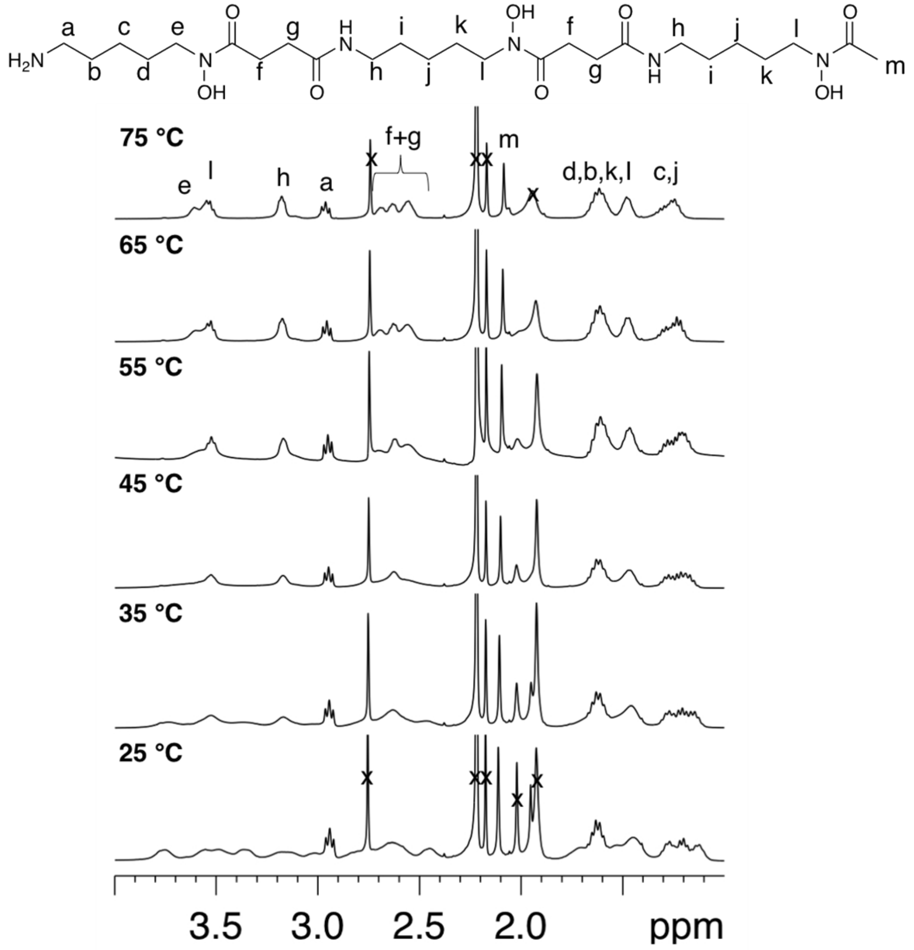

Figure S2. Variable temperature ^1H NMR spectra of $[Zr(DFO)]^+$ in deuterium oxide. Acetone, mesylate counter ion, excess zirconium acetylacetonate and unbound acetylacetone marked (×).

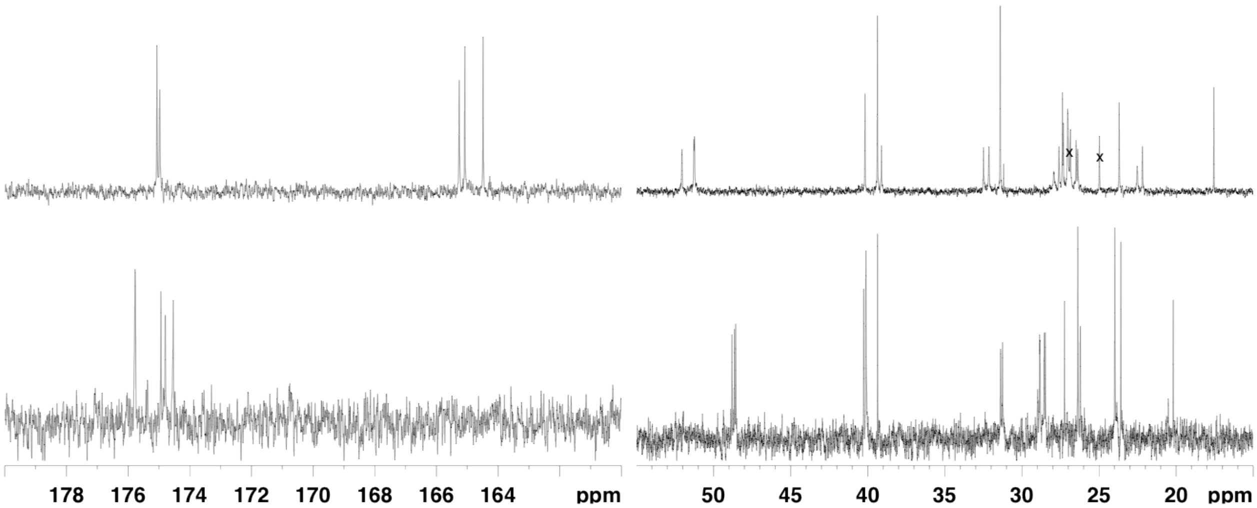

Figure S3. ^{13}C NMR spectra of H_3DFO (bottom) and $[Zr(DFO)]^+$ (top) in deuterium oxide at 25 °C. Chemical shifts are similar to those observed in methanol-*d_4*. Excess zirconium acetylacetonate and unbound acetylacetone marked (×).

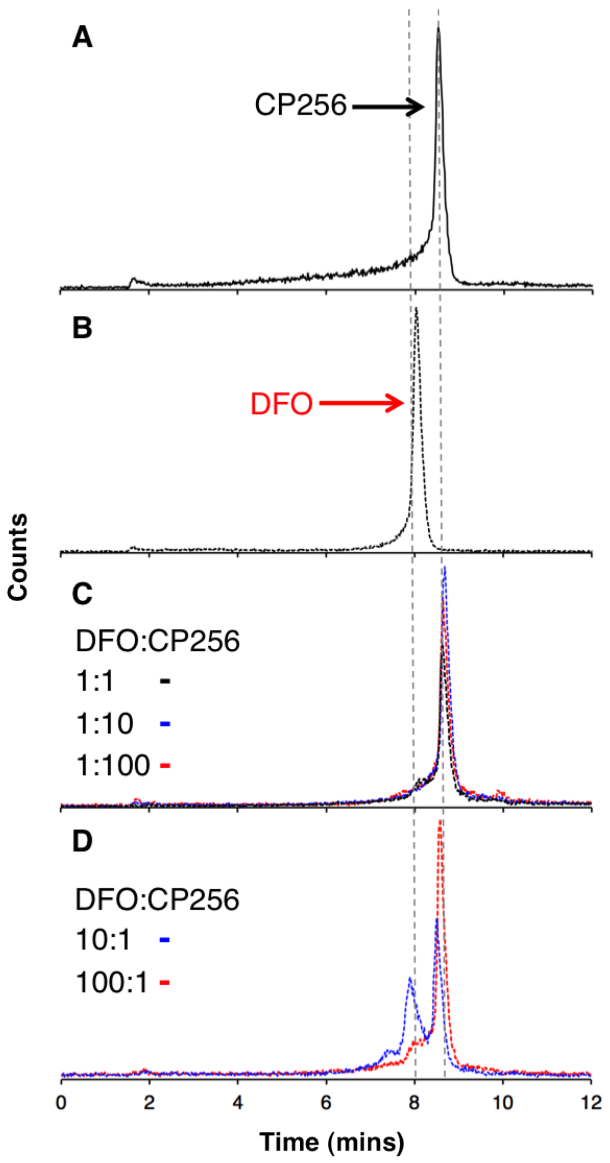

Figure S4. Reverse phase HPLC radiochromatograms of H_3DFO/H_3CP256 competition studies for $^{89}Zr^{4+}$: Reverse phase radiochromatogram of (**A**) $[^{89}Zr(CP256)]^+$ (black) and (**B**) $[^{89}Zr(DFO)]^+$ (dotted line); (**C**) A solution of $[^{89}Zr(DFO)]^+$ incubated with H_3CP256 ligand ($H_3DFO:H_3CP256$ = 1:1, black line); ($H_3DFO:H_3CP256$ = 1:10, blue line); ($H_3DFO:H_3CP256$ = 1:100, red line); in all experiments, final $[H_3DFO]$ = 500 μM. (**D**) A solution of $[^{89}Zr(DFO)]^+$ incubated with H_3CP256 ligand ($H_3DFO:H_3CP256$ = 10:1, red line); ($H_3DFO:H_3CP256$ = 100:1, blue line); in all experiments, final $[H_3CP256]$ = 500 μM.

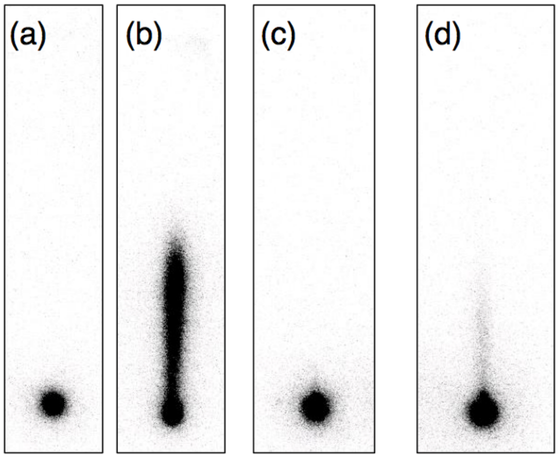

Figure S5. Phosphorimages of ITLC strips of (a) $[^{89}Zr(CP256)]^{+}$; (b) $[^{89}Zr(DFO)]^{+}$; Solution products formed from reaction of $[^{89}Zr(DFO)]^{+}$ with H_3CP256 under conditions where (c) H_3CP256:DFO = 1 and (d) H_3CP256:DFO = 0.1.

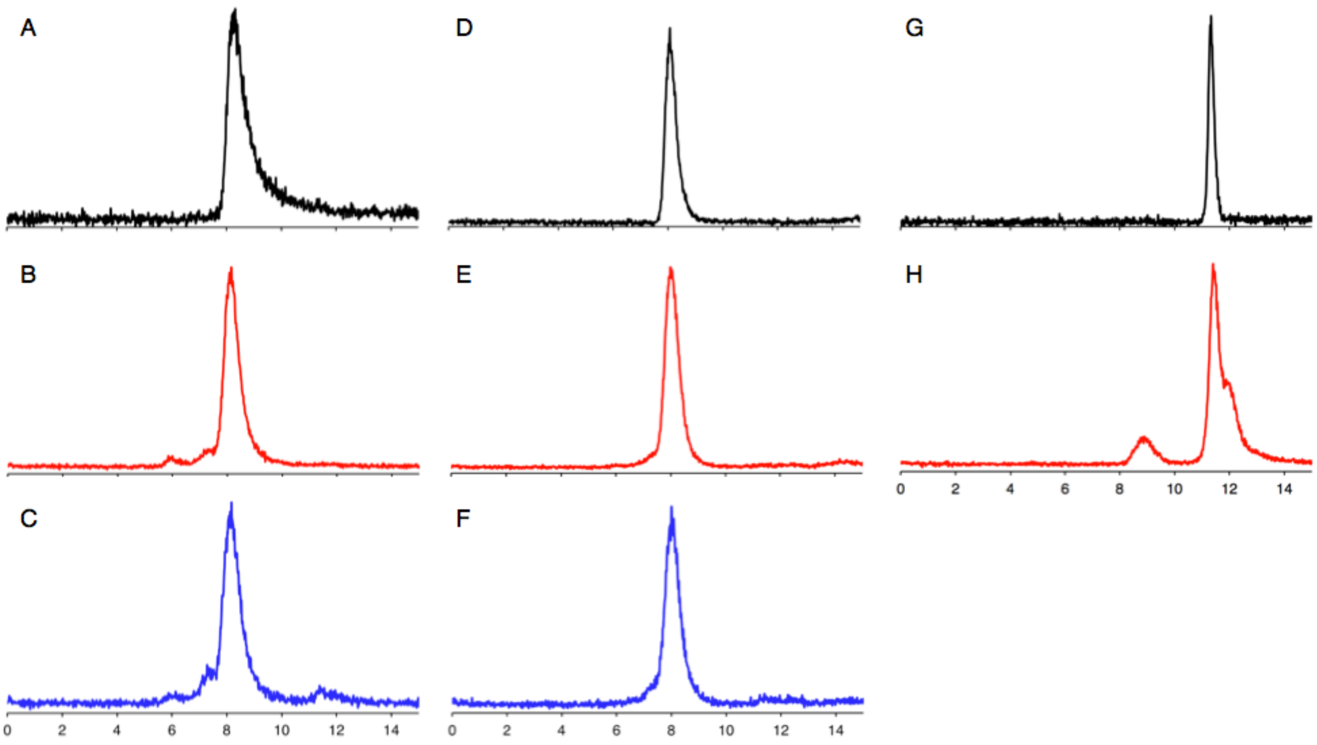

Figure S6. Size exclusion HPLC radiochromatograms of trastuzumab conjugates and serum stability studies: Size exclusion HPLC radiochromatograms of: ^{89}Zr-YM103-trastuzumab without serum (**A**), and incubated in serum at 37 °C for 48 h (**B**) and 168 h (**C**); ^{89}Zr-DFO-trastuzumab without serum (**D**), and incubated in serum at 37 °C for 48 h (**E**) and 168 h (**F**); [$^{89}Zr(ox)_4$] without serum (**G**), and incubated in serum for 48 h (**H**). Less than 5 % of $^{89}Zr^{4+}$ is bound to serum proteins for both immunoconjugates after incubation in serum for 168 h.

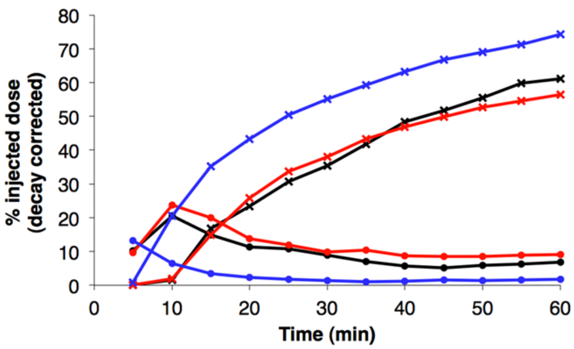

Figure S7. Percentage injected dose of [$^{89}Zr(CP256)]^+$ in kidneys and bladders of three normal male C57Bl/6j mice at 5 – 60 min post-injection: bladder (×), kidneys (•), mouse 1 (black), mouse 2 (red), mouse 3 (blue). In all three mice, [$^{89}Zr(CP256)]^+$ clears rapidly from the blood pool via a renal pathway.
